# Myo10 tail is crucial for promoting long filopodia

**DOI:** 10.1016/j.jbc.2023.105523

**Published:** 2023-12-02

**Authors:** Xingxiang Chen, Jeffrey M. Arciola, Young il Lee, Pak Hung Philip Wong, Haoran Yin, Quanqing Tao, Yuqi Jin, Xianan Qin, H Lee Sweeney, Hyokeun Park

**Affiliations:** 1 Division of Life Science, The Hong Kong University of Science and Technology, Kowloon, Hong Kong, China; 2 Department of Chemistry, University of Florida, Gainesville, USA; 3 Department of Pharmacology & Therapeutics, University of Florida College of Medicine, Gainesville, USA; 4 Department of Physics, The Hong Kong University of Science and Technology, Kowloon, Hong Kong, China; 5 Myology Institute, University of Florida College of Medicine, Gainesville, Florida, USA; 6 State Key Laboratory of Molecular Neuroscience, The Hong Kong University of Science and Technology, Kowloon, Hong Kong, China

**Keywords:** myosin 10, filopodia, coiled coil, dimerization, heavy meromyosin, filopodial elongation

## Abstract

Filopodia are slender cellular protrusions containing parallel actin bundles involved in environmental sensing and signaling, cell adhesion and migration, and growth cone guidance and extension. Myosin 10 (Myo10), an unconventional actin-based motor protein, was reported to induce filopodial initiation with its motor domain. However, the roles of the multifunctional tail domain of Myo10 in filopodial formation and elongation remain elusive. Herein, we generated several constructs of Myo10—full-length Myo10, Myo10 with a truncated tail (Myo10 HMM), and Myo10 containing four mutations to disrupt its coiled-coil domain (Myo10 CC mutant). We found that the truncation of the tail domain decreased filopodial formation and filopodial length, while four mutations in the coiled-coil domain disrupted the motion of Myo10 toward filopodial tips and the elongation of filopodia. Furthermore, we found that filopodia elongated through multiple elongation cycles, which was supported by the Myo10 tail. These findings suggest that Myo10 tail is crucial for promoting long filopodia.

Filopodia are finger-like plasma membrane projections emerging from the actin-rich apices in cells. Actin filaments grow by adding globular actin monomers (G-actin) to its barbed ends at filopodial tips, consequently, inducing the filopodium to outgrow (1). Myosin is a motor protein whose functions are determined by the actin-based motor domain (the “head”) and the versatile “tail” domain (2). Myosin 10 (Myo10) is an unconventional myosin and specifically locates at filopodial tips (3). Structurally, Myo10 comprises the motor head, the neck, and the tail (Fig. 1). The motor domain hydrolyzes ATP and generates force to move along actin filaments. The neck consists of three IQ motifs and a structural single α-helical domain. The Myo10 tail contains a unique structure that starts from the coiled-coil region, which dictates the antiparallel dimerization (4, 5, 6). C-terminal to it is the PEST domain and three PH domains that are involved in recruiting Myo10 to the plasma membrane (7, 8). The MyTH4-FERM domain at the C-terminal end allows Myo10 to regulate cellular mechanotransduction (9, 10, 11, 12). Thus, the Myo10 tail characterizes its unique properties by controlling its interactions with cargoes and the plasma membrane. Disrupting the antiparallel dimerization impaired filopodial induction of Myo10 (4). Overexpression of cargo binding domains (CBDs)–truncated Myo10 did not dramatically affect filopodial induction (3). On the other hand, it was also reported that the motor domain initiated filopodial formation (13). However, the roles of the Myo10 tail in initiating filopodia and regulating filopodial dynamics remain elusive.

**Figure 1:**
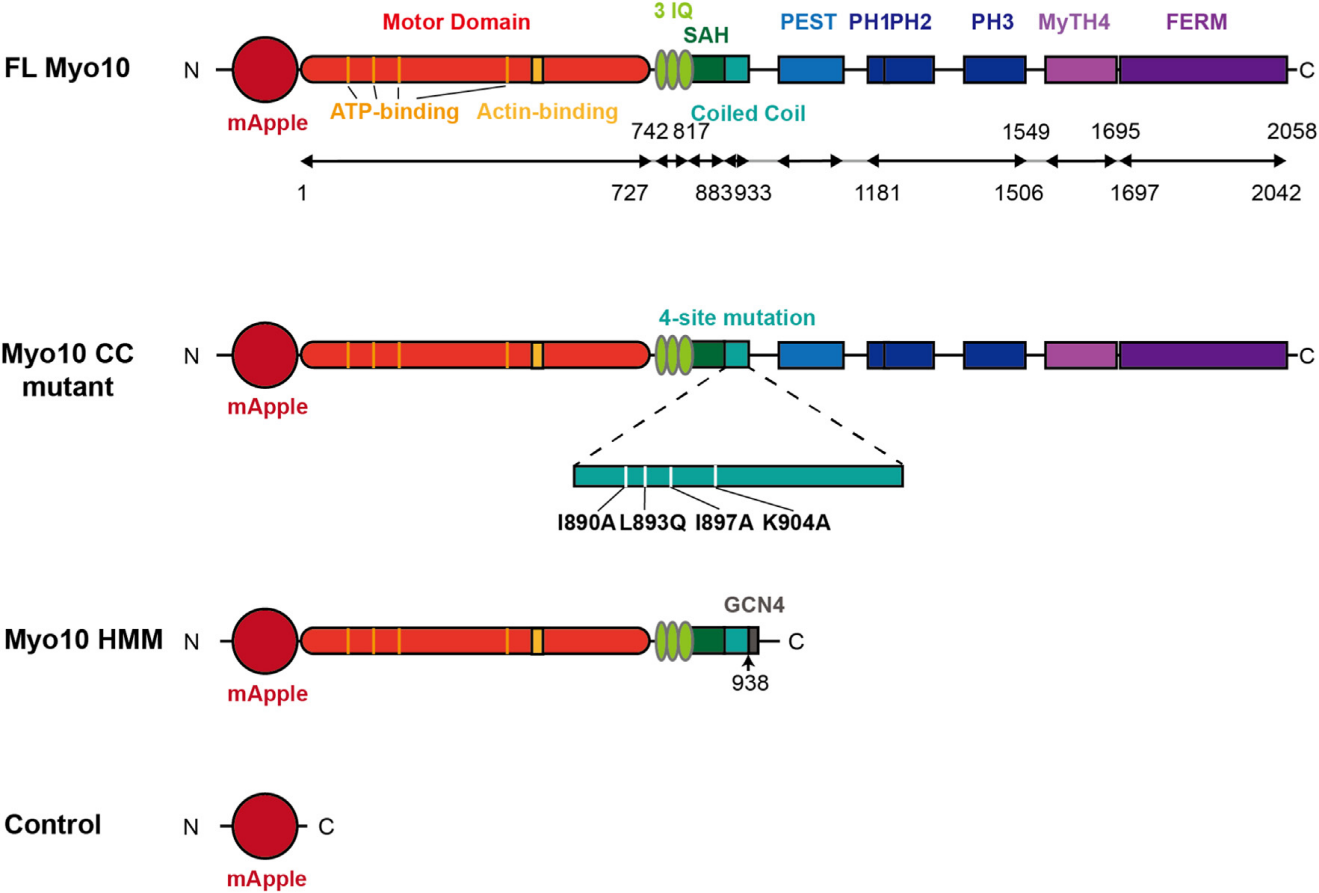
**Schematicillustrates key domains of myosin 10 (Myo10) constructs,including FL Myo10 (aa 1-2058), Myo10 CC mutant (aa 1-2058 with 4 single mutations of I890A, L893Q, I897A, and K904A), Myo10 HMM (aa 1-938), and the empty control (Control) as annotated.** Amino acid numbers and domains of the constructs are indicated. IQ, isoleucine-glutamine motif (or calmodulin binding motif); FERM, Band 4.1, Ezrin, Radixin, Moesin; MyTH4, myosin tail homology 4; PEST, proline, glutamate, serine, and threonine–enriched domain; PH, pleckstrin homology; SAH, single α helix.

In this study, we evaluated the roles of the Myo10 tail in filopodial formation and elongation using several Myo10 constructs. Using real-time live-cell imaging, we visualized filopodial initiation and elongation induced by these Myo10 constructs. We found that either removing the tail or disrupting dimerization decreased the number and length of filopodia. Furthermore, removing the tail or disrupting dimerization of Myo10 prevented filopodia from elongating, whereas full-length Myo10 allowed filopodia to elongate through multiple elongation cycles.

## Results

First, we generated four constructs: a mApple control construct (Control) and three mApple-fused human Myo10 constructs including full-length Myo10 (FL Myo10), tail-truncated Myo10 heavy meromyosin ended with a GCN4 leucine zipper to force dimerization (Myo10 HMM), and coiled-coil four-site mutated Myo10 (Myo10 CC mutant) (Fig. 1). Myo10 forms an antiparallel dimer through hydrophobic and electrostatic interactions in the coiled-coil region, including the residues I890, L893, E894, I897, and K904 (4, 6). We introduced four mutations (I890A, L893Q, I897A, and K904A) to disrupt the antiparallel dimerization interface. These constructs were transfected into COS-7 cells, which have negligible endogenous Myo10 expression (Fig. S1) (3). Exogenous Myo10 expression levels measured by total mApple signal in each COS-7 cell showed no significant difference among mApple-FL-Myo10, mApple-Myo10-HMM, and mApple-Myo10-CC mutant (Fig. S2).

**Figure 2:**
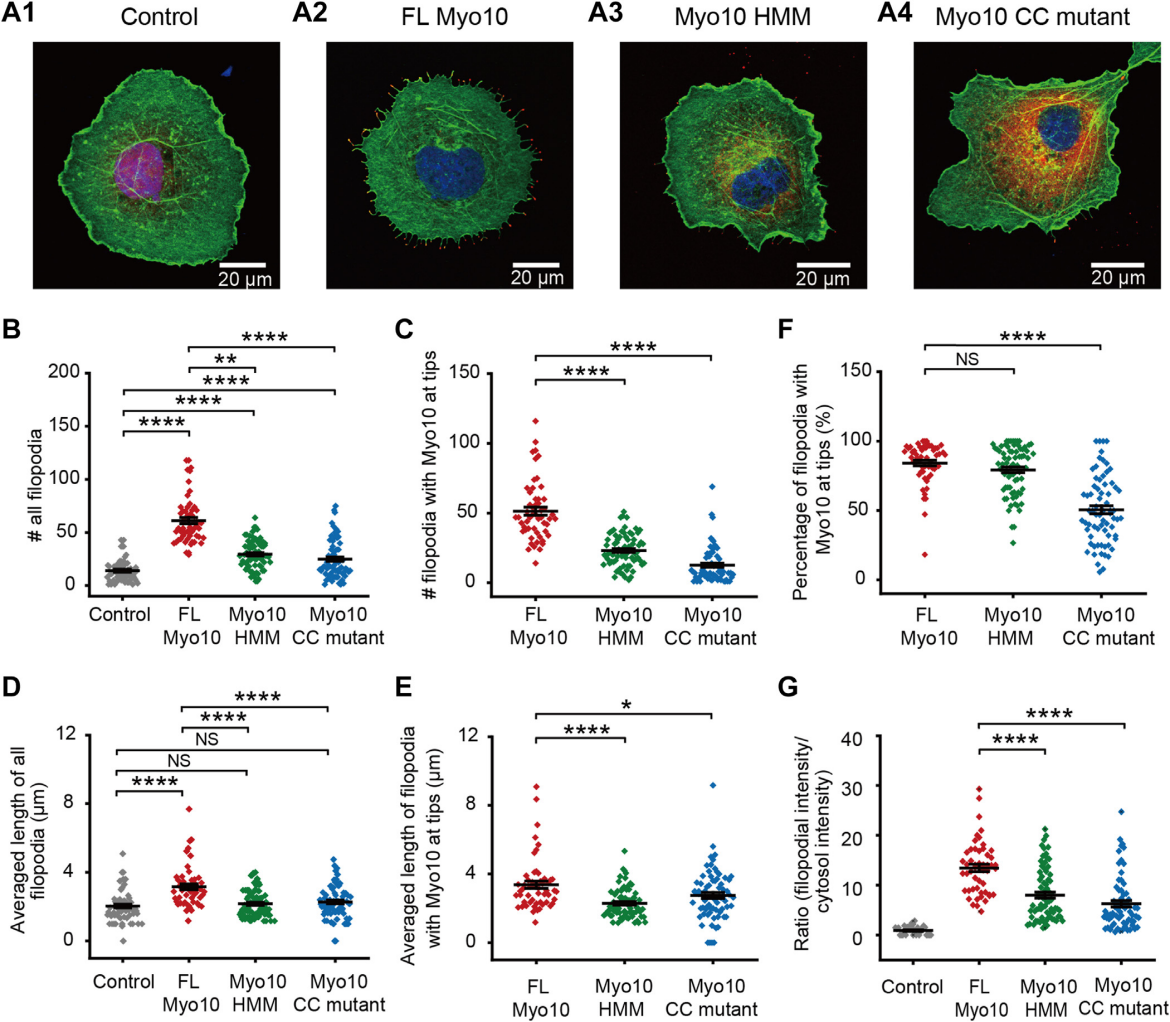
**Roles of Myo10 constructs in filopodial number and length.***A*, representative images of COS-7 cells overexpressing four constructs (A1: Control, A2: FL Myo10, A3: Myo10 HMM, A4: Myo10 CC mutant; *red*) and further stained with phalloidin–Alexa Fluor 488 (*green*) and DAPI (*blue*). Statistical analysis of the number of all filopodia (*B*) and filopodia containing Myo10 at tips (*C*). Statistical quantification of the length of all filopodia (*D*) and filopodia containing Myo10 at tips (*E*). *F*, percentage of Myo10-positive filopodia per cell. *G*, ratio of fluorescence signals of Myo10 at filopodial tips over that in the cytosol. Data were displayed with mean line and whisker (mean ± SEM) plots overlapped with raw data. ∗*p* < 0.05, ∗∗*p* < 0.01, and ∗∗∗∗*p* < 0.0001 (two-sample Student’s *t-*test).

We measured the number of filopodia induced by these constructs in fixed COS-7 cells (Fig. 2, *A* and *B*). FL Myo10 generated the largest number of filopodia per cell (61.2 ± 3.02) (average ± standard error of the mean [SEM], N = 54 cells) (Fig. 2*B*). Myo10 HMM (29. 5 ± 1.60, N = 73) or Myo10 CC mutant (24.8 ± 2.12, N = 71) induced fewer filopodia compared with FL Myo10 but more filopodia than Control (14.0 ± 1.40, N = 64) (Fig. 2*B*). These results suggest that both the motor and tail domain of Myo10 contribute to the formation of filopodia. Compared with Control (2.04 ± 0.11 μm), significantly longer filopodia were induced by FL Myo10 (3.16 ± 0.16 μm) but neither by Myo10 HMM (2.16 ± 0.09 μm) nor by Myo10 CC mutant (2.27 ± 0.11 μm) (Fig. 2*D*), suggesting the Myo10 tail plays an important role in the elongation of filopodia.

To further understand roles of the Myo10 tail in the formation and elongation of filopodia, the spatial distribution of exogenously expressed Myo10 was examined by calculating the fluorescence ratio of Myo10 between filopodial tips and the cytosol (see details in Experimental procedures). FL Myo10 was mostly located at filopodial tips (ratio = 13.5 ± 0.73, N = 52) (Fig. 2*G*), whereas Myo10 HMM was more sparsely dispersed in the cytosol (ratio = 8.0 ± 0.61, N = 74). Myo10 CC mutant showed significantly decreased localization at filopodial tips and increased cytosolic localization (ratio = 6.3 ± 0.64, N = 68) (Fig. 2*G*). These results suggest that proper dimerization is important for Myo10 localization at filopodial tips. We further tested whether this mislocalization is related to the filopodial generation. Each cell expressing one of these constructs was divided into two subgroups based on the fluorescence intensity ratio. We found that the high level of mislocalized Myo10 CC mutant in the cytosol was closely correlated with decreased capacity to induce and elongate filopodia (Fig. S3).

We next measured the percentage of Myo10-positive filopodia. Most filopodia induced by FL Myo10 (84.2 ± 2.07%, N = 54) and Myo10 HMM (79.3 ± 2.11%, N = 73) contained Myo10 at tips (Fig. 2*F*), indicating that dimerization of Myo10 facilitates its localization at filopodial tips, consistent with previous reports (4, 14). In contrast, only half of filopodia induced by Myo10 CC mutant contained Myo10 at tips (50.51 ± 2.88%, N = 54), suggesting that disrupting proper dimerization hinders Myo10 from moving to filopodial tips. To understand how Myo10 proteins at filopodial tips affect the formation and elongation of filopodia, we further counted the number of filopodia containing Myo10 at tips. The number of filopodia containing Myo10 HMM at tips (23.2 ± 1.37, N = 73) in cells expressing Myo10 HMM was smaller than that with FL Myo10 (51.4 ± 2.90, N = 54) (Fig. 2*C*) but was almost double that with Myo10 CC mutant (12.7 ± 1.48, N = 71) (Fig. 2*C*). The length of filopodia containing Myo10 HMM (2.29 ± 0.10 μm) or Myo10 CC mutant (2.74 ± 0.17 μm) at tips was shorter than that of filopodia with FL Myo10 at tips (3.37 ± 0.21 μm) (Fig. 2*E*), consistent with the results of all filopodia (Fig. 2*D*). These results confirm that the Myo10 tail plays a critical role in filopodial formation and elongation.

**Figure 3:**
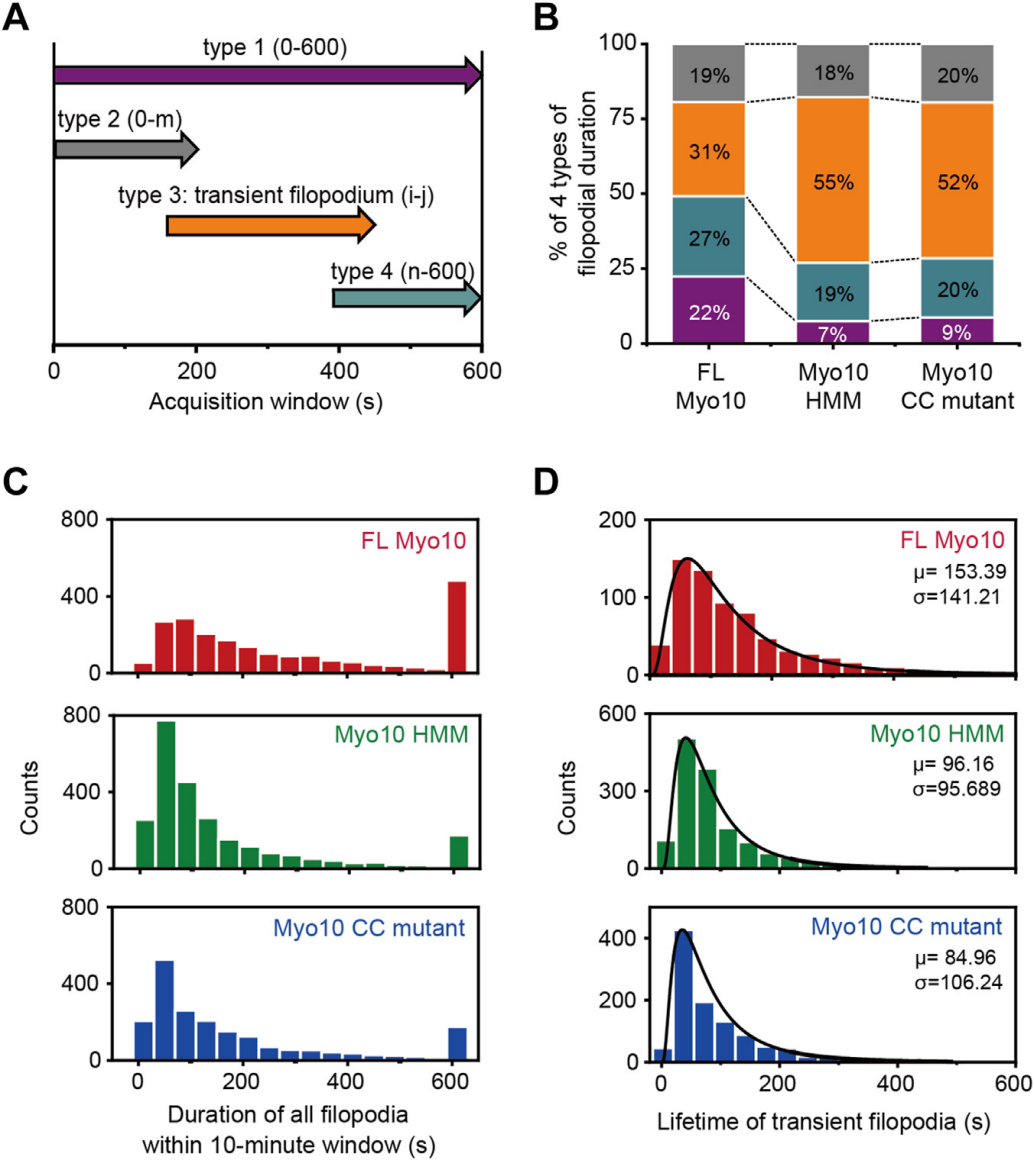
**FL Myo10 induces longer-lived filopodia.***A*, filopodia were categorized into four types based on time of appearance and disappearance within the 10-min acquisition time window. *B*, percentage of four types of filopodia. The percentage was annotated on the fraction of individual types. *C*, duration histogram of individual filopodia with a bin size of 40. *D*, lifetime histogram of transient filopodia with a bin size of 35, which was further fitted with a lognormal distribution function (*black curves*). Fitting values for the histogram of FL Myo10 filopodia (*red*): R^2^ = 0.99. Fitting values for the histogram of Myo10 HMM filopodia (*green*): R^2^ = 0.98. Fitting values for the histogram of Myo10 CC mutant filopodia (*blue*): R^2^ = 0.99.

To further investigate the detailed roles of Myo10 tail, we conducted real-time imaging of filopodia (Movies S1–S3) and observed four distinct types of filopodia based on their appearance and disappearance during the 10-min observation time—type 1 filopodia remained existed during the imaging period; type 2 filopodia appeared before the onset of imaging and disappeared during imaging; type 3 filopodia appeared and disappeared during imaging; type 4 filopodia appeared during imaging and remained until the end of imaging (Fig. 3*A*). We quantified the percentage of each type of filopodia induced by Myo10 constructs. FL Myo10 induced the largest percentage of long-lasting (type 1) filopodia (22%) and the lowest percentage of transient (type 3) filopodia (31%) among three Myo10 constructs, whereas Myo10 HMM induced the smallest portion of long-lasting filopodia (7%) and the largest portion of transient filopodia (55%) (Fig. 3*B*). Myo10 CC mutant showed a similar trend as Myo10 HMM: 9% of long-lasting filopodia and 52% of transient filopodia (Fig. 3*B*). Moreover, we calculated the duration of filopodia within the 10-min observation period. Duration histograms of filopodia showed that FL Myo10-induced filopodia dwelled longer than those induced by Myo10 HMM or Myo10 CC mutant (Fig. 3*C*). We further analyzed the lifetime of transient (type 3) filopodia, which appeared and disappeared during the imaging period. Lifetime histograms were fitted with a single lognormal distribution (Fig. 3*D*). FL Myo10-induced transient filopodia (average [*μ*] = 153.39 s) has a longer lifetime than those induced by Myo10 HMM (*μ* = 96.16 s) or Myo10 CC mutant (*μ* = 84.96 s). Thus, our results indicate that the Myo10 tail plays a crucial role in increasing the lifetime of filopodia.

**Figure 4:**
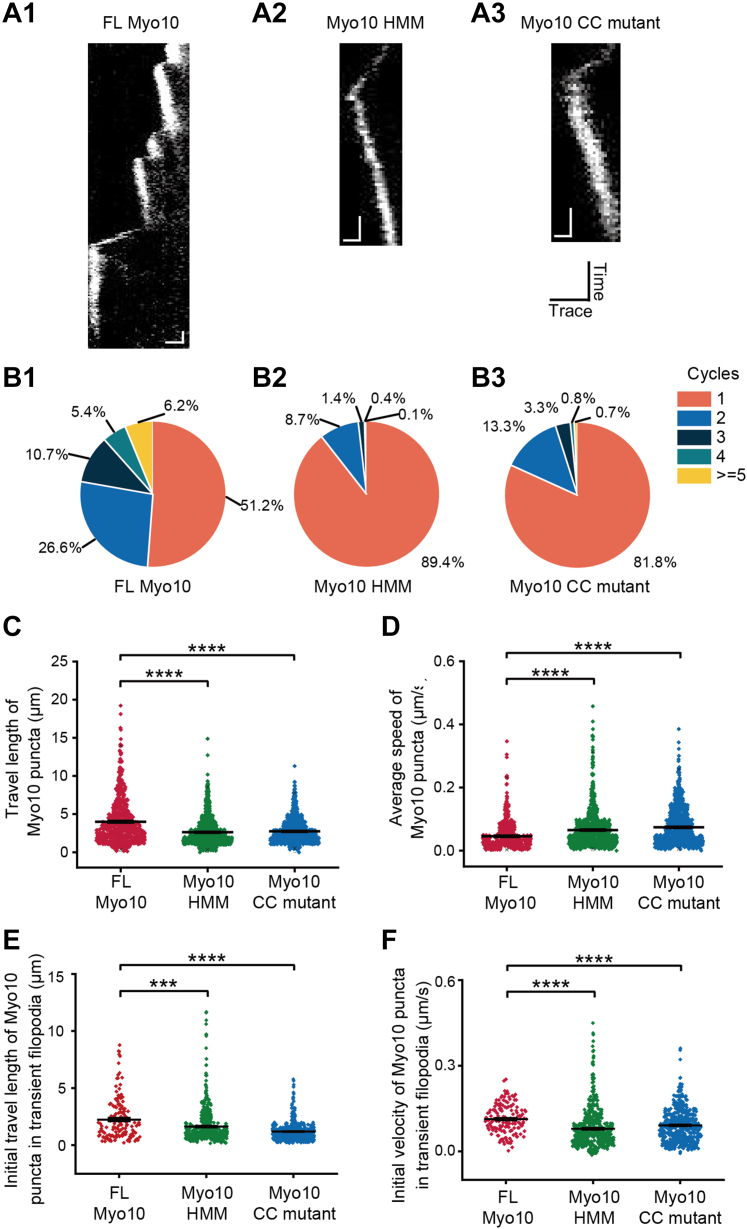
**FL Myo10induces filopodia elongation *via* multiple extension–retraction cycles.***A*, representative kymographs along path with respect to time for filopodia capped with FL Myo10, Myo10 HMM, and Myo10 CC mutant, respectively. The horizontal scale bar on kymograph represents 1 μm and vertical scale bar represents 30 s. *B*, percentages of elongation cycles in each filopodium within a 10-min acquisition time. Different colors represent different number of cycles. *C*, travel length of Myo10 puncta at filopodial tips. *D*, average speed of Myo10 puncta at filopodial tips. Travel length (*E*) and velocity (*F*) of Myo10 puncta in transient filopodia (type 3) in their first elongation cycle. Data were displayed with mean line and whisker (mean ± SEM) plots overlapped with raw data. ∗∗∗*p* < 0.001, ∗∗∗∗*p* < 0.0001 (two-sample Student’s *t*-test).

Furthermore, we analyzed the dynamics of filopodia by monitoring Myo10 puncta at tips using Kymolyzer (Fig. 4*A*). We observed multiple extension–retraction cycles in FL Myo10–induced filopodia (Fig. 4*B*); around half filopodia (48.8%) induced by FL Myo10 showed more than one extension–retraction cycle within the observation period. In contrast, we observed significantly fewer extension–retraction cycles of filopodia in cells expressing Myo10 HMM or Myo10 CC mutant (10.6% for Myo10 HMM and 18.2% for Myo10 CC mutant). These results suggest that the Myo10 tail induces filopodial elongation through multiple rounds of extension–retraction cycles. Moreover, we analyzed the travel length and average speed of Myo10 puncta at filopodial tips. The travel length of FL Myo10 puncta at filopodial tips (4.00 ± 0.12 μm, n = 665 filopodia) was longer than that of Myo10 HMM (2.62 ± 0.06 μm, n = 1024) or Myo10 CC mutant puncta (2.74 ± 0.05 μm, n = 913) (Fig. 4*C*). Average speed of FL Myo10 puncta at filopodial tips (45.7 ± 2.19 nm/s) was lower than that of Myo10 HMM (65.18 ± 1.78 nm/s) or Myo10 CC mutant (74.09 ± 1.92 nm/s) (Fig. 4*D*). The lower average speed of FL Myo10 puncta at filopodial tips might be correlated with the longer duration of FL Myo10 induced filopodia (Fig. 3, *C* and *D*). To examine this, we excluded the stalling segments and further examined the speed of filopodial elongation and retraction. Filopodia with FL Myo10 elongated with the fastest speed (164.9 ± 4.82 nm/s) compared with those with Myo10 HMM (99.7 ± 2.88 nm/s) and Myo10 CC mutant (103.8 ± 2.62 nm/s) (Fig. S4*A*). On the contrary, filopodia with FL Myo10 retracted with the lowest speed (35.9 ± 1.08 nm/s) compared with those with Myo10 HMM (43.5 ± 1.35 nm/s) and Myo10 CC mutant (45.8 ± 1.19 nm/s) (Fig. S4*B*). Finally, we calculated the travel length and velocity of Myo10 puncta in the first extension cycle of type 3 transient filopodia. The travel length of FL Myo10 puncta in the first extension cycle of transient filopodia (2.26 ± 0.15 μm, n = 154 filopodia) was longer than that of Myo10 HMM (1.65 ± 0.07 μm, n = 600) or Myo10 CC mutant puncta (1.19 ± 0.03 μm, n = 572) (Fig. 4*E*). The initial velocity of Myo10 puncta in the first extension cycle of transient filopodia in expressing FL Myo10 (112.9 ± 4.04 nm/s) was higher than that of Myo10 HMM (78.9 ± 2.94 nm/s) or Myo10 CC mutant puncta (91.0 ± 2.41 nm/s) (Fig. 4*F*), indicating that the disrupted tail domain decreases the capability of Myo10 to induce long filopodia. Taken together, our results suggest that Myo10 tail plays an essential role in elongating filopodia through multiple rounds of elongation–retraction cycles.

## Discussion

Myo10 has a unique tail that plays multiple roles (2, 10, 15, 16, 17, 18). In this study, we showed that tail-truncated Myo10 HMM retained its ability to form a dimer and move to distal tips of filopodia but failed to induce as many and long filopodia as FL Myo10. In contrast, Myo10 CC mutant not only was mislocalized in the cytosol but also decreased its capability to form and elongate filopodia. Thus, these results suggest that Myo10 tail is crucial for forming and elongating filopodia.

Our phalloidin staining results showed more filopodia formed in cells expressing FL Myo10 or Myo10 HMM compared with cells expressing the control construct (Fig. 2*B*), implying that the intact motor activity can initiate filopodia (13, 19). Moreover, FL Myo10 induced more filopodia than Myo10 HMM (Fig. 2, *B* and *C*), suggesting that Myo10 tail also facilitates filopodial formation. Real-time imaging of filopodia revealed that filopodia induced by Myo10 HMM or Myo10 CC mutant were more transient, implying that removing the Myo10 tail decreases the capability of Myo10 to retain formed filopodia. Retaining formed filopodia is necessary for elongation of filopodia, which is the consequence of the elongation of actin filaments and the adhesion of protrusions to matrices against the retrograde flow and cell plasma membrane contractility (20, 21, 22). Removing the Myo10 tail is likely to disrupt membrane binding, cargo binding and transport, including the transport of Mena/VASP (an actin polymerization regulator), and interaction with focal adhesions through integrins (11, 12, 18, 23, 24, 25, 26).

Our real-time imaging of filopodia also showed that filopodia elongated through multiple cycles of elongation–retraction. FL Myo10–induced filopodia elongated faster and retracted slower than tail-truncated Myo10 HMM and tail-mutated Myo10 CC mutant (Fig. S4). After fast elongation, filopodia experienced slow retraction prior to another round of elongation. However, Myo10 HMM failed to induce multiple rounds of elongation, resulting in the formation of shorter filopodia (Fig. 2, *D* and *E*). Moreover, the initial velocity and elongation length of transient filopodia induced by Myo10 HMM were slower and shorter than those induced by FL Myo10 (Fig. 4, *E* and *F*). Therefore, our results suggest that Myo10 HMM retains the capability to form a dimer and move to distal filopodial tips but fails to induce multiple elongation cycles and to produce long filopodia, further confirming that the Myo10 tail is critical for forming long filopodia (25, 26). Furthermore, significant number of Myo10 CC mutant was located in the cytosol and failed to move to filopodial tips (Fig. 2, *F* and *G*), indicating that the coiled-coil domain of Myo10 plays an important role in its proper localization and the formation of long filipodia (Fig. S3). Some Myo10 CC mutant proteins were located at filopodia tips (Fig. 2, *F* and *G*), but the removal of CBDs in Myo10 CC mutant (Myo10 CC mutant truncate) completely prevented the localization of Myo10 at filopodial tips (Fig. S5), which implies that the CBDs may allow some Myo10 CC mutant to move to filopodial tips. Myo10 CC mutant also induced fewer and shorter filopodia than FL Myo10 but more filopodia compared with the construct with mApple only (Fig. 2*B*), suggesting that the intact motor domain is needed for inducing filopodia formation and that proper dimerization of Myo10 is crucial for optimal induction and elongation of filopodia.

How the Myo10 tail induces filopodial formation and elongation remains to be determined. Recent single-molecule motility assays of Myo10 revealed unique stepping behaviors of Myo10, which are closely related to its antiparallel coiled-coil region (6, 27, 28, 29, 30). Therefore, further research is needed to elucidate the detailed roles of unique features of Myo10 in formation and elongation of filopodia.

In summary, we showed that the Myo10 tail plays a crucial role in formation and elongation of filopodia. Particularly, the Myo10 tail is essential for forming long filopodia through multiple rounds of extension–retraction cycles. Thus, our results provide new insights into the roles of the domains of the motor protein in cellular functions.

## Experimental procedures

### Cell culture

COS-7 cells (ATCC, CRL-1651) were purchased from ATCC and used between passages 3 and 15. Cells were cultured in a 37 °C incubator supplied with 5% CO_2_ in Dulbecco's modified Eagle’s medium (Gibco, 12100-061) with 10% (v/v) fetal bovine serum (Gibco No. 10270-106) and 1% (v/v) penicillin–streptomycin (10,000 U mL^−1^, Gibco, 15114022). COS-7 cells were passaged when cell confluency reached 80 to 90%.

### Immunocytochemistry

COS-7 cells were passaged in six-well plates overnight and transfected with mApple-Myo10 plasmids with lipofectamine 2000 (Invitrogen, 11668019) according to the manufacturer’s protocol. After overnight incubation, cells were subcultured on coverslips (18 mm, No. 0) coated with 50 μg mL^-1^ Poly-D-lysine (Sigma-Aldrich, P7405). Cells were ready for phalloidin staining on the following day. Briefly, cells were rinsed twice with PBS, fixed in 4% (w/v) paraformaldehyde (PFA-PBS, pH 6.9, Sigma-Aldrich, P6148) for 15 min at room temperature, and washed three times with PBS (10 min per wash). Then cells were permeabilized and blocked with 0.1% Triton-X 100-PBS supplied with 1% bovine serum albumin for another 15 min at room temperature. Actin filaments were stained with Phalloidin-Alexa Fluor 488 (Invitrogen, A12379) in PBS with 0.1% Triton-X 100 and 1% bovine serum albumin for 50 min. DAPI (Invitrogen, D1306) was added and incubated for another 10 min for staining nuclei. The coverslip was washed 3 times before being mounted to glass slides with HYDROMOUNT (National Diagnostics, NAT1324) for confocal imaging. Leica SP8 confocal microscope was used for checking the morphology of COS-7 cells overexpressing exogenous mApple-Myo10, including mApple-FL Myo10, mApple-HMM Myo10, mApple-CC mutant Myo10, Myo10 CC mutant truncate, and mApple only. Lasers of 405 nm, 488 nm, and 552 nm were sequentially used to excite DAPI, Alexa Fluor 488 conjugated phalloidin, and mApple, respectively.

### Analyzing filopodium from individual cells

To quantify the length and number of filopodia, images were processed and analyzed in Fiji (an open source software). Only the peripheral filopodia, which were attached onto the coverslip surface, rather than dorsal filopodia, were used for analyses. First, three channels were merged with identical colors. We then measured the length of each filopodium and counted the number of filopodia for individual cells. The intensity ratio of filopodium over cytoplasm was analyzed using a custom-made python program. Briefly, images were preprocessed with Gaussian blurring to reduce noise, followed by segmentation of cell edges by triangle thresholding and hole filling. Filopodium was then differentiated and split by erosion and dilation. Myo10 signals at filopodial tips were measured using local minimum thresholding and used to calculate the ratio of Myo10 signal at filopodial tips over that in the cytosol using Equation 1 in the below. Cells were further divided into two subgroups of high ratio (ratio score ≥ 8) and low ratio (ratio score < 8). The number and length of filopodia were further compared between high ratio subgroups and low ratio subgroups.

(1) $$Ratio=\frac{\frac{sum\;of\;fluorescence\;intensity\;in\;each\;filopodium}{number\;of\;pixels\;containing\;nonzero\;intenstity\;in\;thefilopodium\;}\;}{\frac{sum\;of\;fluorescence\;intensity\;in\;the\;cytosol}{number\;of\;pixels\;in\;the\;cytosol}}$$

### Live-cell imaging

COS-7 cells were seeded on glass-bottomed dishes overnight and then transfected with mApple-Myo10 and LifeAct-GFP using lipofectamine 2000 (Invitrogen, 11668019). After overnight incubation, dishes were mounted onto a 37 °C chamber and supplied with 5% CO_2_. Imaging was performed in a growth medium using Leica TCS SP8 inverted confocal microscope with an oil-immersion objective (NA = 1.40, 63×). An integrated 488-nm laser and a 552-nm laser were used to excite LifeAct-GFP and mApple-fused Myo10, respectively. Fluorescence images (1024 × 1024 pixels, 300 frames) were acquired with a scanning speed of 600 Hz and an acquisition frequency of 0.5 Hz.

### Mapping filopodial motility from living cells

To map the dynamic elongation and retraction of filopodia, images were first processed in Fiji and data were analyzed using Kymolyzer (a macro program) to automate commands and extract the motility parameters describing the filopodial dynamics (31). Briefly, maximum projections over the duration of each filopodium were generated, followed by highlighting the central axis of the track path centripetally. Kymograph was then created by projecting the pixel-based intensity on the path axis against time. Next, tips identified by accumulated mApple–Myo10 signals were tracked by selecting objects consecutively along the time axis on kymographs. Duration (recorded existing time during the 10-min acquisition window) and cycle (extension–retraction cycles) were obtained directly from kymographs. Finally, parameters, including travel length, speed, and velocity, were extracted to decipher the dynamics of individual filopodia.

## Data availability

All relevant data can be found within the article and the supporting information that includes a supporting figure, supporting movies, and the sequences of all DNA constructs.

## Supporting information

This article contains supporting information.

## Conflict of interest

The authors declare that they have no conflicts of interest with the contents of this article.
