# Supplementary material for: Myo10 tail is crucial for promoting long filopodia
Source: J Biol Chem. 2023 Dec 2;300(1):105523. doi: 10.1016/j.jbc.2023.105523 (PMC10790087; doi:10.1016/j.jbc.2023.105523)
Supplement: Supporting information [file mmc4.docx]

**Myo10 tail is crucial for promoting long filopodia**

Xingxiang Chen^1^, [Jeffrey M Arciola](https://pubmed.ncbi.nlm.nih.gov/?size=100&term=Arciola+JM&cauthor_id=29907615)^2,+^, Young il Lee^3^, Pak Hung Philip Wong^4^, Haoran Yin^1^, Quanqing Tao^4^, Yuqi Jin^4^, Xianan Qin^4, ‡^, H Lee Sweeney^3,5,*^ and Hyokeun Park^1,4,6,*^

^1^Division of Life Science, The Hong Kong University of Science and Technology, Clear Water Bay, Kowloon, Hong Kong

^2^Department of Chemistry, University of Florida, Gainesville, United States

^3^Department of Pharmacology & Therapeutics, University of Florida College of Medicine, Gainesville, United States

^4^Department of Physics, The Hong Kong University of Science and Technology,

Clear Water Bay, Kowloon, Hong Kong

^5^University of Florida Myology Institute, Gainesville, United States

^6^State Key Laboratory of Molecular Neuroscience, The Hong Kong University of Science and Technology, Clear Water Bay, Kowloon, Hong Kong

List of supporting information:

1. Figure S1.
2. Figure S2.
3. Figure S3.
4. Figure S4.
5. Figure S5.
6. Sequence of the constructs used in this work:
7. mApple-FL Myo10
8. mApple-Myo10 CC mutant
9. mApple-Myo10 HMM
10. mApple-Control
11. mApple-Myo10 CC mutant truncate

7. Movies

a. Movie S1. The Dynamic of filopodia induced by FL Myo10.

b. Movie S2. The Dynamic of filopodia induced by Myo10 HMM.

c. Movie S3. The Dynamic of filopodia induced by Myo10 CC mutant.


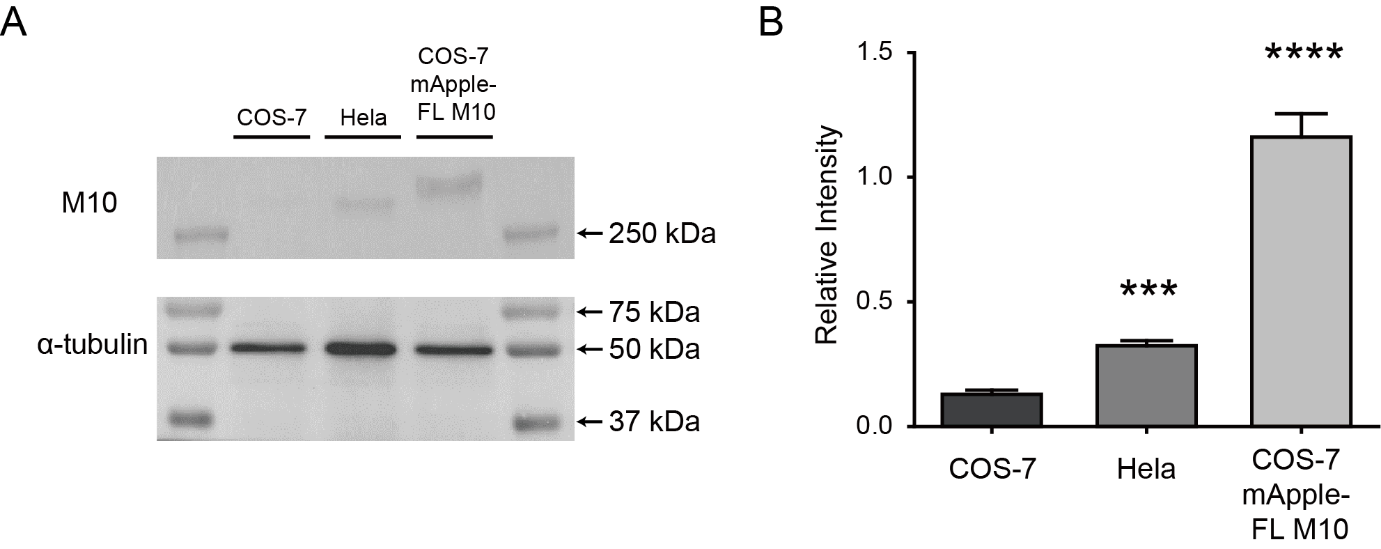


**Figure S1.** Expression level of endogenous Myo10 proteins in COS-7 cells is low. **A.** Immunoblotting of endogenous Myo10 proteins in COS-7 cells (lane 2), Hela cells (lane 3), and exogenously mApple-FL Myo10 expressing COS-7 cells (lane 4). The molecular-weight of protein ladder (Lane 1 and Lane 5) was marked by arrows. **B.** The expression levels of Myo10 (Mean ± SEM) were quantified (n = 3 independent cultures; normalized to α-tubulin). Two-sample Student’s *t*-test was used for statistical analyses. ****p* < 0.001 and *****p* < 0.0001).


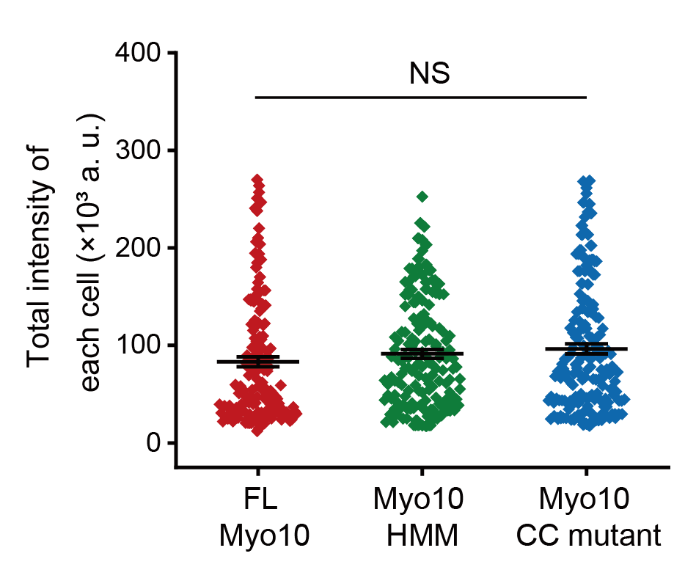


**Figure S2.** Expression levels of exogeneous Myo10 constructs in COS-7 cells were similar. COS-7 cells were transfected with the same amount of plasmid DNA of mApple-FL-Myo10, mApple-Myo10-HMM, and mApple-Myo10-CC mutant. Expression levels were evaluated by measuring the total fluorescence signals of mApple in every transfected cell (4 independent cultures). Two-sample Student’s *t*-test was used for statistical analyses. Data was displayed with mean line and whisker (mean ± SEM) plots overlapped with raw data. NS: not significant.


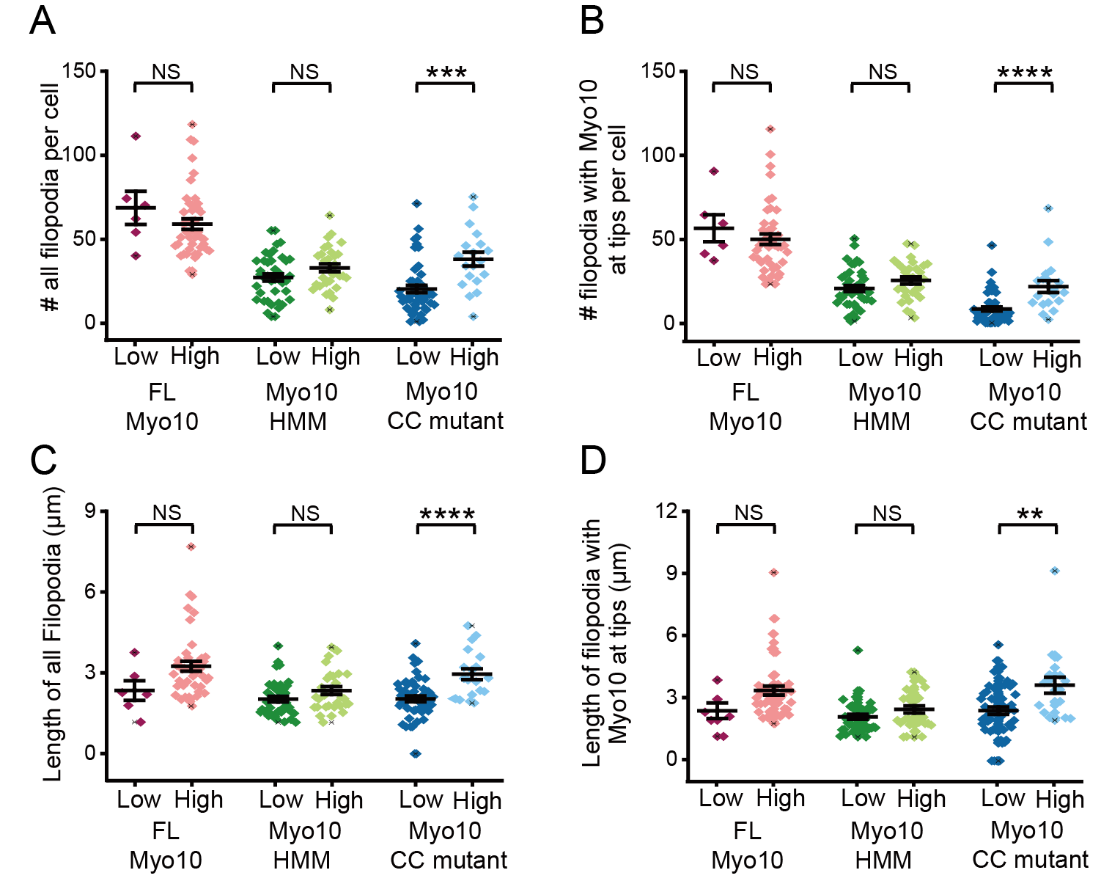


**Figure S3.** The level of mislocalized M10 CC mutant is correlated with decreased capability to induce more and longer filopodia. Transfected cells were divided into two subgroups of high ratio and low ratio based on a threshold fluorescence signal ratio between filopodial tips and cytosol of 8. A total of 50 cells expressing FL Myo10 were divided into two subgroups with low ratio (6 cells, deep red) and high ratio (44 cells, pink). 42% of Myo10 HMM expressing cells (30 cells, dark green) showed a low ratio while the rest of them showed a high ratio subclass (41 cells, lime green). Most Myo10 CC mutant expressing cells showed low ratio (51 cells, yale blue). The number and the length of all filopodia (**A** and **C**) and the filopodia containing Myo10 signals at the tips (**B** and **D**) were calculated. Both the number and length of filopodia in cells having low ratios were significantly lower and shorter than cells having high ratio (19 cells, sky blue) in Myo10 CC mutant group. Two-sample Student’s *t*-test was used for statistical analyses. Data was displayed with mean line and whisker (mean ± SEM) plots overlapped with raw data. **p* < 0.05, ***p* < 0.01, ****p* < 0.001 and **** *p* < 0.0001.


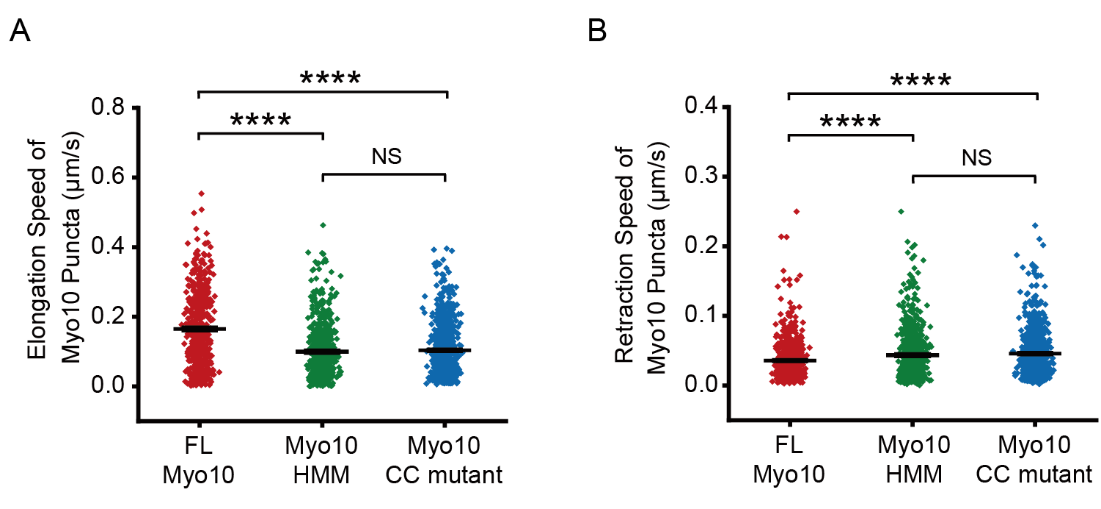


**Figure S4.** Elongation (**A**) and retraction (**B**) speed of filopodial tips marked by fluorescence signals of Myo10. Filopodia induced by FL Myo10 elongated with the fastest speed, while retracting with the lowest speed (Mean ± S.D.). Two-sample Student’s *t*-test was used for statistical analyses. Data was displayed with mean line and whisker (mean ± SEM) plots overlapped with raw data. *****p* < 0.0001 and NS: not significant.


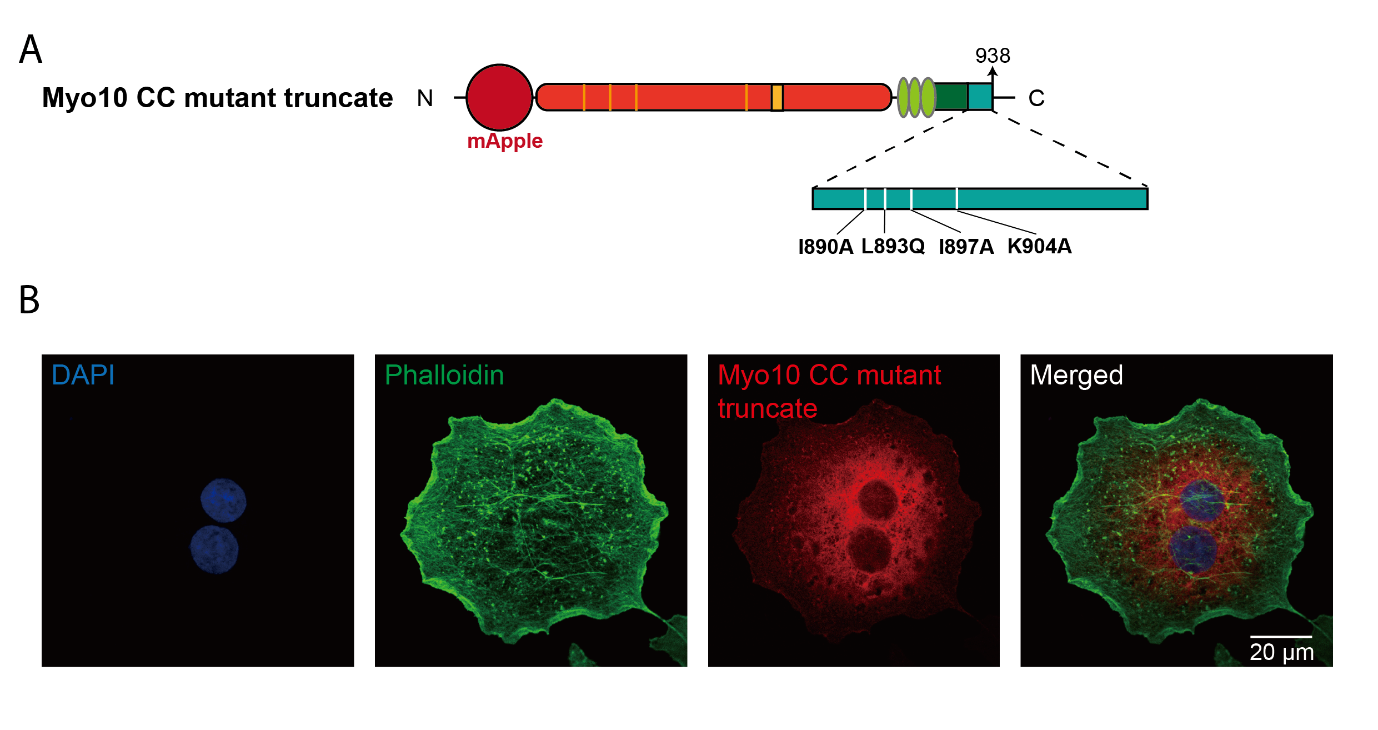


**Figure S5.** Myo10 CC mutant truncate without cargo-binding domains failed to localize to the tips of filopodia. **A.** This construct was truncated at site of aa938 to generate mApple-Myo10 CC truncate. **B.** Phalloidin staining of COS-7 cell expressed mApple-Myo10 CC mutant truncate.

**Sequence of the constructs used in this work**

1. **mApple-FL Myo10**

size: 12405 bp

cloning sites: 5’ – NotI & 3’ – XbaI

CMV promoter

KOZAK

FLAG-tag

mApple

FL-MyoX

bGH PA terminator

GACGGATCGGGAGATCTCCCGATCCCCTATGGTGCACTCTCAGTACAATCTGCTCTGATGCCGCATAGTTAAGCCAGTATCTGCTCCCTGCTTGTGTGTTGGAGGTCGCTGAGTAGTGCGCGAGCAAAATTTAAGCTACAACAAGGCAAGGCTTGACCGACAATTGCATGAAGAATCTGCTTAGGGTTAGGCGTTTTGCGCTGCTTCGCGATGTACGGGCCAGATATACGCGTTGACATTGATTATTGACTAGTTATTAATAGTAATCAATTACGGGGTCATTAGTTCATAGCCCATATATGGAGTTCCGCGTTACATAACTTACGGTAAATGGCCCGCCTGGCTGACCGCCCAACGACCCCCGCCCATTGACGTCAATAATGACGTATGTTCCCATAGTAACGCCAATAGGGACTTTCCATTGACGTCAATGGGTGGAGTATTTACGGTAAACTGCCCACTTGGCAGTACATCAAGTGTATCATATGCCAAGTACGCCCCCTATTGACGTCAATGACGGTAAATGGCCCGCCTGGCATTATGCCCAGTACATGACCTTATGGGACTTTCCTACTTGGCAGTACATCTACGTATTAGTCATCGCTATTACCATGGTGATGCGGTTTTGGCAGTACATCAATGGGCGTGGATAGCGGTTTGACTCACGGGGATTTCCAAGTCTCCACCCCATTGACGTCAATGGGAGTTTGTTTTGGCACCAAAATCAACGGGACTTTCCAAAATGTCGTAACAACTCCGCCCCATTGACGCAAATGGGCGGTAGGCGTGTACGGTGGGAGGTCTATATAAGCAGAGCTCTCTGGCTAACTAGAGAACCCACTGCTTACTGGCTTATCGAAATTAATACGACTCACTATAGGGAGACCCAAGCTGGCTAGTTAAGCTTGGTACCGAGCTCGGATCCACTAGTCCAGTGTGGTGGAATTCTGCAGATATCCAGCACAGTGGCGGCCgccaccATGgactacaaggacgatgacgacaagGGCGTGAGCAAGGGCGAGGAGAATAACATGGCCATCATCAAGGAGTTCATGCGCTTCAAGGTGCACATGGAGGGCTCCGTGAACGGCCACGAGTTCGAGATCGAGGGCGAGGGCGAGGGCCGCCCCTACGAGGCCTTTCAGACCGCTAAGCTGAAGGTGACCAAGGGTGGCCCCCTGCCCTTCGCCTGGGACATCCTGTCCCCTCAGTTCATGTACGGCTCCAAGGTCTACATTAAGCACCCAGCCGACATCCCCGACTACTTCAAGCTGTCCTTCCCCGAGGGCTTCAGGTGGGAGCGCGTGATGAACTTCGAGGACGGCGGCATTATTCACGTTAACCAGGACTCCTCCCTGCAGGACGGCGTGTTCATCTACAAGGTGAAGCTGCGCGGCACCAACTTCCCCTCCGACGGCCCCGTAATGCAGAAGAAGACCATGGGCTGGGAGGCCTCCGAGGAGCGGATGTACCCCGAGGACGGCGCCCTGAAGAGCGAGATCAAGAAGAGGCTGAAGCTGAAGGACGGCGGCCACTACGCCGCCGAGGTCAAGACCACCTACAAGGCCAAGAAGCCCGTGCAGCTGCCCGGCGCCTACATCGTCGACATCAAGTTGGACATCGTGTCCCACAACGAGGACTACACCATCGTGGAACAGTACGAACGCGCCGAGGGCCGCCACTCCACCGGCGGCATGGACGAGCTGTACAAGggcggccgaGATAACTTCTTCACCGAGGGAACACGGGTCTGGCTGAGAGAAAATGGCCAGCATTTTCCAAGTACTGTAAATTCCTGTGCAGAAGGCATCGTCGTCTTCCGGACAGACTATGGTCAGGTATTCACTTACAAGCAGAGCACAATTACCCACCAGAAGGTGACTGCTATGCACCCCACGAACGAGGAGGGCGTGGATGACATGGCGTCCTTGACAGAGCTCCATGGCGGCTCCATCATGTATAACTTATTCCAGCGGTATAAGAGAAATCAAATATATACCTACATCGGCTCCATCCTGGCCTCTGTGAACCCCTACCAGCCCATCGCCGGGCTGTACGAGCCTGCCACCATGGAGCAGTACAGCCGGCGCCACCTGGGCGAGCTGCCCCCGCACATCTTCGCCATCGCCAACGAGTGCTACCGCTGCCTGTGGAAGCGCCACGACAACCAGTGCATCCTCATCAGTGGTGAAAGTGGGGCAGGTAAAACCGAAAGCACTAAATTGATCCTCAAGTTTCTGTCAGTCATCAGTCAACAGTCTTTGGAATTGTCCTTAAAGGAGAAGACATCCTGTGTTGAACGAGCTATTCTTGAAAGCAGCCCCATCATGGAAGCTTTCGGCAATGCGAAGACCGTGTACAACAACAACTCTAGTCGCTTTGGGAAGTTTGTTCAGCTGAACATCTGTCAGAAAGGAAATATTCAGGGCGGGAGAATTGTAGATTATTTATTAGAAAAAAACCGAGTAGTAAGGCAAAATCCCGGGGAAAGGAATTATCACATATTTTATGCACTGCTGGCAGGGCTGGAACATGAAGAAAGAGAAGAATTTTATTTATCTACGCCAGAAAACTACCACTACTTGAATCAGTCTGGATGTGTAGAAGACAAGACAATCAGTGACCAGGAATCCTTTAGGGAAGTTATTACGGCAATGGACGTGATGCAGTTCAGCAAGGAGGAAGTTCGGGAAGTGTCGAGGCTGCTTGCTGGTATACTGCATCTTGGGAACATAGAATTTATCACTGCTGGTGGGGCACAGGTTTCCTTCAAAACAGCTTTGGGCAGATCTGCGGAGTTACTTGGGCTGGACCCAACACAGCTCACAGATGCTTTGACCCAGAGATCAATGTTCCTCAGGGGAGAAGAGATCCTCACGCCTCTCAATGTTCAACAGGCAGTAGACAGCAGGGACTCCCTGGCCATGGCTCTGTATGCGTGCTGCTTTGAGTGGGTAATCAAGAAGATCAACAGCAGGATCAAAGGCAATGAGGACTTCAAGTCTATTGGCATCCTCGACATCTTTGGATTTGAAAACTTTGAGGTTAATCACTTTGAACAGTTCAATATAAACTATGCAAACGAGAAACTTCAGGAGTACTTCAACAAGCATATTTTTTCTTTAGAACAACTAGAATATAGCCGGGAAGGATTAGTGTGGGAAGATATTGACTGGATAGACAATGGAGAATGCCTGGACTTGATTGAGAAGAAACTTGGCCTCCTAGCCCTTATCAATGAAGAAAGCCATTTTCCTCAAGCCACAGACAGCACCTTATTGGAGAAGCTACACAGTCAGCATGCGAATAACCACTTTTATGTGAAGCCCAGAGTTGCAGTTAACAATTTTGGAGTGAAGCACTATGCTGGAGAGGTGCAATATGATGTCCGAGGTATCTTGGAGAAGAACAGAGATACATTTCGAGATGACCTTCTCAATTTGCTAAGAGAAAGCCGATTTGACTTTATCTACGATCTTTTTGAACATGTTTCAAGCCGCAACAACCAGGATACCTTGAAATGTGGAAGCAAACATCGGCGGCCTACAGTCAGCTCACAGTTCAAGGACTCACTGCATTCCTTAATGGCAACGCTAAGCTCCTCTAATCCTTTCTTTGTTCGCTGTATCAAGCCAAACATGCAGAAGATGCCAGACCAGTTTGACCAGGCGGTTGTGCTGAACCAGCTGCGGTACTCAGGGATGCTGGAGACTGTGAGAATCCGCAAAGCTGGGTATGCGGTCCGAAGACCCTTTCAGGACTTTTACAAAAGGTATAAAGTGCTGATGAGGAATCTGGCTCTGCCTGAGGACGTCCGAGGGAAGTGCACGAGCCTGCTGCAGCTCTATGATGCCTCCAACAGCGAGTGGCAGCTGGGGAAGACCAAGGTCTTTCTTCGAGAATCCTTGGAACAGAAACTGGAGAAGCGGAGGGAAGAGGAAGTGAGCCACGCGGCCATGGTGATTCGGGCCCATGTCTTGGGCTTCTTAGCACGAAAACAATACAGAAAGGTCCTTTATTGTGTGGTGATAATACAGAAGAATTACAGAGCATTCCTTCTGAGGAGGAGATTTTTGCACCTGAAAAAGGCAGCCATAGTTTTCCAGAAGCAACTCAGAGGTCAGATTGCTCGGAGAGTTTACAGACAATTGCTGGCAGAGAAAAGGGAGCAAGAAGAAAAGAAGAAACAGGAAGAGGAAGAAAAGAAGAAACGGGAGGAAGAAGAAAGAGAAAGAGAGAGAGAGCGAAGAGAAGCCGAGCTCCGCGCCCAGCAGGAAGAAGAAACGAGGAAGCAGCAAGAACTCGAAGCCTTGCAGAAGAGCCAGAAGGAAGCTGAACTGACCCGTGAACTGGAGAAACAGAAGGAAAATAAGCAGGTGGAAGAGATCCTCCGTCTGGAGAAAGAAATCGAGGACCTGCAGCGCATGAAGGAGCAGCAGGAGCTGTCGCTGACCGAGGCTTCCCTGCAGAAGCTGCAGGAGCGGCGGGACCAGGAGCTCCGCAGGCTGGAGGAGGAAGCGTGCAGGGCGGCCCAGGAGTTCCTCGAGTCCCTCAATTTCGACGAGATCGACGAGTGTGTCCGGAATATCGAGCGGTCCCTGTCGGTGGGAAGCGAATTTTCCAGCGAGCTGGCTGAGAGCGCATGCGAGGAGAAGCCCAACTTCAACTTCAGCCAGCCCTACCCAGAGGAGGAGGTCGATGAGGGCTTCGAAGCCGACGACGACGCCTTCAAGGACTCCCCCAACCCCAGCGAGCACGGCCACTCAGACCAGCGAACAAGTGGCATCCGGACCAGCGATGACTCTTCAGAGGAGGACCCATACATGAACGACACGGTGGTGCCCACCAGCCCCAGTGCGGACAGCACGGTGCTGCTCGCCCCATCAGTGCAGGACTCCGGGAGCCTACACAACTCCTCCAGCGGCGAGTCCACCTACTGCATGCCCCAGAACGCTGGGGACTTGCCCTCCCCAGACGGCGACTACGACTACGACCAGGATGACTATGAGGACGGTGCCATCACTTCCGGCAGCAGCGTGACCTTCTCCAACTCCTACGGCAGCCAGTGGTCCCCCGACTACCGCTGCTCTGTGGGGACCTACAACAGCTCGGGTGCCTACCGGTTCAGCTCTGAGGGGGCGCAGTCCTCGTTTGAAGATAGTGAAGAGGACTTTGATTCCAGGTTTGATACAGATGATGAGCTTTCATACCGGCGTGACTCTGTGTACAGCTGTGTCACTCTGCCGTATTTCCACAGCTTTCTGTACATGAAAGGTGGCCTGATGAACTCTTGGAAACGCCGCTGGTGCGTCCTCAAGGATGAAACCTTCTTGTGGTTCCGCTCCAAGCAGGAGGCCCTCAAGCAAGGCTGGCTCCACAAAAAAGGGGGGGGCTCCTCCACGCTGTCCAGGAGAAATTGGAAGAAGCGCTGGTTTGTCCTCCGCCAGTCCAAGCTGATGTACTTTGAAAACGACAGCGAGGAGAAGCTCAAGGGCACCGTAGAAGTGCGAACGGCAAAAGAGATCATAGATAACACCACCAAGGAGAATGGGATCGACATCATTATGGCCGATAGGACTTTCCACCTGATTGCAGAGTCCCCAGAAGATGCCAGCCAGTGGTTCAGCGTGCTGAGTCAGGTCCACGCGTCCACGGACCAGGAGATCCAGGAGATGCATGATGAGCAGGCAAACCCACAGAATGCTGTGGGCACCTTGGATGTGGGGCTGATTGATTCTGTGTGTGCCTCTGACAGCCCTGATAGACCCAACTCGTTTGTGATCATCACGGCCAACCGGGTGCTGCACTGCAACGCCGACACGCCGGAGGAGATGCACCACTGGATAACCCTGCTGCAGAGGTCCAAAGGGGACACCAGAGTGGAGGGCCAGGAATTCATCGTGAGAGGATGGTTGCACAAAGAGGTGAAGAACAGTCCAAAGATGTCTTCACTGAAACTGAAGAAACGGTGGTTTGTACTCACCCACAATTCCCTGGATTACTACAAGAGTTCAGAGAAGAACGCGCTCAAACTGGGGACCCTGGTCCTCAACAGCCTCTGCTCTGTCGTCCCCCCAGATGAGAAGATATTCAAAGAGACAGGCTACTGGAACGTCACCGTGTACGGGCGCAAGCACTGTTACCGGCTCTACACCAAGCTGCTCAACGAGGCCACCCGGTGGTCCAGTGCCATTCAAAACGTGACTGACACCAAGGCCCCGATCGACACCCCCACCCAGCAGCTGATTCAAGATATCAAGGAGAACTGCCTGAACTCGGATGTGGTGGAACAGATTTACAAGCGGAACCCGATCCTTCGATACACCCATCACCCCTTGCACTCCCCGCTCCTGCCCCTTCCGTATGGGGACATAAATCTCAACTTGCTCAAAGACAAAGGCTATACCACCCTTCAGGATGAGGCCATCAAGATATTCAATTCCCTGCAGCAACTGGAGTCCATGTCTGACCCAATTCCAATAATCCAGGGCATCCTACAGACAGGGCATGACCTGCGACCTCTGCGGGACGAGCTGTACTGCCAGCTTATCAAACAGACCAACAAAGTGCCCCACCCCGGCAGTGTGGGCAACCTGTACAGCTGGCAGATCCTGACATGCCTGAGCTGCACCTTCCTGCCGAGTCGAGGGATTCTCAAGTATCTCAAGTTCCATCTGAAAAGGATACGGGAACAGTTTCCAGGAACCGAGATGGAAAAATACGCTCTCTTCACTTACGAATCTCTTAAGAAAACCAAATGCCGAGAGTTTGTGCCTTCCCGAGATGAAATAGAAGCTCTGATCCACAGGCAGGAAATGACATCCACGGTCTATTGCCATGGCGGCGGCTCCTGCAAGATCACCATCAACTCCCACACCACCGCTGGGGAGGTGGTGGAGAAGCTGATCCGAGGCCTGGCCATGGAGGACAGCAGGAACATGTTTGCTTTGTTTGAATACAACGGCCACGTCGACAAAGCCATTGAAAGTCGAACCGTCGTAGCTGATGTCTTAGCCAAGTTTGAAAAGCTGGCTGCCACATCCGAGGTTGGGGACCTGCCATGGAAATTCTACTTCAAACTTTACTGCTTCCTGGACACAGACAACGTGCCAAAAGACAGTGTGGAGTTTGCATTTATGTTTGAACAGGCCCACGAAGCGGTTATCCATGGCCACCATCCAGCCCCGGAAGAAAACCTCCAGGTTCTTGCTGCCCTGCGACTCCAGTATCTGCAGGGGGATTATACTCTGCACGCTGCCATCCCACCTCTCGAAGAGGTTTATTCCCTGCAGAGACTCAAGGCCCGCATCAGCCAGTCAACCAAAACCTTCACCCCTTGTGAACGGCTGGAGAAGAGGCGGACGAGCTTCCTAGAGGGGACCCTGAGGCGGAGCTTCCGGACAGGATCCGTGGTCCGGCAGAAGGTCGAGGAGGAGCAGATGCTGGACATGTGGATTAAGGAAGAAGTCTCCTCTGCTCGAGCCAGTATCATTGACAAGTGGAGGAAATTTCAGGGAATGAACCAGGAACAGGCCATGGCCAAGTACATGGCCTTGATCAAGGAGTGGCCTGGCTATGGCTCGACGCTGTTTGATGTGGAGTGCAAGGAAGGTGGCTTCCCTCAGGAACTCTGGTTGGGTGTCAGCGCGGACGCCGTCTCCGTCTACAAGCGTGGAGAGGGAAGACCACTGGAAGTCTTCCAGTATGAACACATCCTCTCTTTTGGGGCACCCCTGGCGAATACGTATAAGATCGTGGTCGATGAGAGGGAGCTGCTCTTTGAAACCAGTGAGGTGGTGGATGTGGCCAAGCTCATGAAAGCCTACATCAGCATGATCGTGAAGAAGCGCTACAGCACGACACGCTCCGCCAGCAGCCAGGGCAGCTCCAGGTAATCTAGAGGGCCCTTCGAACAAAAACTCATCTCAGAAGAGGATCTGAATATGCATACCGGTCATCATCACCATCACCATTGAGTTTAAACCCGCTGATCAGCCTCGACTGTGCCTTCTAGTTGCCAGCCATCTGTTGTTTGCCCCTCCCCCGTGCCTTCCTTGACCCTGGAAGGTGCCACTCCCACTGTCCTTTCCTAATAAAATGAGGAAATTGCATCGCATTGTCTGAGTAGGTGTCATTCTATTCTGGGGGGTGGGGTGGGGCAGGACAGCAAGGGGGAGGATTGGGAAGACAATAGCAGGCATGCTGGGGATGCGGTGGGCTCTATGGCTTCTGAGGCGGAAAGAACCAGCTGGGGCTCTAGGGGGTATCCCCACGCGCCCTGTAGCGGCGCATTAAGCGCGGCGGGTGTGGTGGTTACGCGCAGCGTGACCGCTACACTTGCCAGCGCCCTAGCGCCCGCTCCTTTCGCTTTCTTCCCTTCCTTTCTCGCCACGTTCGCCGGCTTTCCCCGTCAAGCTCTAAATCGGGGGCTCCCTTTAGGGTTCCGATTTAGTGCTTTACGGCACCTCGACCCCAAAAAACTTGATTAGGGTGATGGTTCACGTAGTGGGCCATCGCCCTGATAGACGGTTTTTCGCCCTTTGACGTTGGAGTCCACGTTCTTTAATAGTGGACTCTTGTTCCAAACTGGAACAACACTCAACCCTATCTCGGTCTATTCTTTTGATTTATAAGGGATTTTGCCGATTTCGGCCTATTGGTTAAAAAATGAGCTGATTTAACAAAAATTTAACGCGAATTAATTCTGTGGAATGTGTGTCAGTTAGGGTGTGGAAAGTCCCCAGGCTCCCCAGCAGGCAGAAGTATGCAAAGCATGCATCTCAATTAGTCAGCAACCAGGTGTGGAAAGTCCCCAGGCTCCCCAGCAGGCAGAAGTATGCAAAGCATGCATCTCAATTAGTCAGCAACCATAGTCCCGCCCCTAACTCCGCCCATCCCGCCCCTAACTCCGCCCAGTTCCGCCCATTCTCCGCCCCATGGCTGACTAATTTTTTTTATTTATGCAGAGGCCGAGGCCGCCTCTGCCTCTGAGCTATTCCAGAAGTAGTGAGGAGGCTTTTTTGGAGGCCTAGGCTTTTGCAAAAAGCTCCCGGGAGCTTGTATATCCATTTTCGGATCTGATCAAGAGACAGGATGAGGATCGTTTCGCATGATTGAACAAGATGGATTGCACGCAGGTTCTCCGGCCGCTTGGGTGGAGAGGCTATTCGGCTATGACTGGGCACAACAGACAATCGGCTGCTCTGATGCCGCCGTGTTCCGGCTGTCAGCGCAGGGGCGCCCGGTTCTTTTTGTCAAGACCGACCTGTCCGGTGCCCTGAATGAACTGCAGGACGAGGCAGCGCGGCTATCGTGGCTGGCCACGACGGGCGTTCCTTGCGCAGCTGTGCTCGACGTTGTCACTGAAGCGGGAAGGGACTGGCTGCTATTGGGCGAAGTGCCGGGGCAGGATCTCCTGTCATCTCACCTTGCTCCTGCCGAGAAAGTATCCATCATGGCTGATGCAATGCGGCGGCTGCATACGCTTGATCCGGCTACCTGCCCATTCGACCACCAAGCGAAACATCGCATCGAGCGAGCACGTACTCGGATGGAAGCCGGTCTTGTCGATCAGGATGATCTGGACGAAGAGCATCAGGGGCTCGCGCCAGCCGAACTGTTCGCCAGGCTCAAGGCGCGCATGCCCGACGGCGAGGATCTCGTCGTGACCCATGGCGATGCCTGCTTGCCGAATATCATGGTGGAAAATGGCCGCTTTTCTGGATTCATCGACTGTGGCCGGCTGGGTGTGGCGGACCGCTATCAGGACATAGCGTTGGCTACCCGTGATATTGCTGAAGAGCTTGGCGGCGAATGGGCTGACCGCTTCCTCGTGCTTTACGGTATCGCCGCTCCCGATTCGCAGCGCATCGCCTTCTATCGCCTTCTTGACGAGTTCTTCTGAGCGGGACTCTGGGGTTCGAAATGACCGACCAAGCGACGCCCAACCTGCCATCACGAGATTTCGATTCCACCGCCGCCTTCTATGAAAGGTTGGGCTTCGGAATCGTTTTCCGGGACGCCGGCTGGATGATCCTCCAGCGCGGGGATCTCATGCTGGAGTTCTTCGCCCACCCCAACTTGTTTATTGCAGCTTATAATGGTTACAAATAAAGCAATAGCATCACAAATTTCACAAATAAAGCATTTTTTTCACTGCATTCTAGTTGTGGTTTGTCCAAACTCATCAATGTATCTTATCATGTCTGTATACCGTCGACCTCTAGCTAGAGCTTGGCGTAATCATGGTCATAGCTGTTTCCTGTGTGAAATTGTTATCCGCTCACAATTCCACACAACATACGAGCCGGAAGCATAAAGTGTAAAGCCTGGGGTGCCTAATGAGTGAGCTAACTCACATTAATTGCGTTGCGCTCACTGCCCGCTTTCCAGTCGGGAAACCTGTCGTGCCAGCTGCATTAATGAATCGGCCAACGCGCGGGGAGAGGCGGTTTGCGTATTGGGCGCTCTTCCGCTTCCTCGCTCACTGACTCGCTGCGCTCGGTCGTTCGGCTGCGGCGAGCGGTATCAGCTCACTCAAAGGCGGTAATACGGTTATCCACAGAATCAGGGGATAACGCAGGAAAGAACATGTGAGCAAAAGGCCAGCAAAAGGCCAGGAACCGTAAAAAGGCCGCGTTGCTGGCGTTTTTCCATAGGCTCCGCCCCCCTGACGAGCATCACAAAAATCGACGCTCAAGTCAGAGGTGGCGAAACCCGACAGGACTATAAAGATACCAGGCGTTTCCCCCTGGAAGCTCCCTCGTGCGCTCTCCTGTTCCGACCCTGCCGCTTACCGGATACCTGTCCGCCTTTCTCCCTTCGGGAAGCGTGGCGCTTTCTCATAGCTCACGCTGTAGGTATCTCAGTTCGGTGTAGGTCGTTCGCTCCAAGCTGGGCTGTGTGCACGAACCCCCCGTTCAGCCCGACCGCTGCGCCTTATCCGGTAACTATCGTCTTGAGTCCAACCCGGTAAGACACGACTTATCGCCACTGGCAGCAGCCACTGGTAACAGGATTAGCAGAGCGAGGTATGTAGGCGGTGCTACAGAGTTCTTGAAGTGGTGGCCTAACTACGGCTACACTAGAAGAACAGTATTTGGTATCTGCGCTCTGCTGAAGCCAGTTACCTTCGGAAAAAGAGTTGGTAGCTCTTGATCCGGCAAACAAACCACCGCTGGTAGCGGTTTTTTTGTTTGCAAGCAGCAGATTACGCGCAGAAAAAAAGGATCTCAAGAAGATCCTTTGATCTTTTCTACGGGGTCTGACGCTCAGTGGAACGAAAACTCACGTTAAGGGATTTTGGTCATGAGATTATCAAAAAGGATCTTCACCTAGATCCTTTTAAATTAAAAATGAAGTTTTAAATCAATCTAAAGTATATATGAGTAAACTTGGTCTGACAGTTACCAATGCTTAATCAGTGAGGCACCTATCTCAGCGATCTGTCTATTTCGTTCATCCATAGTTGCCTGACTCCCCGTCGTGTAGATAACTACGATACGGGAGGGCTTACCATCTGGCCCCAGTGCTGCAATGATACCGCGAGACCCACGCTCACCGGCTCCAGATTTATCAGCAATAAACCAGCCAGCCGGAAGGGCCGAGCGCAGAAGTGGTCCTGCAACTTTATCCGCCTCCATCCAGTCTATTAATTGTTGCCGGGAAGCTAGAGTAAGTAGTTCGCCAGTTAATAGTTTGCGCAACGTTGTTGCCATTGCTACAGGCATCGTGGTGTCACGCTCGTCGTTTGGTATGGCTTCATTCAGCTCCGGTTCCCAACGATCAAGGCGAGTTACATGATCCCCCATGTTGTGCAAAAAAGCGGTTAGCTCCTTCGGTCCTCCGATCGTTGTCAGAAGTAAGTTGGCCGCAGTGTTATCACTCATGGTTATGGCAGCACTGCATAATTCTCTTACTGTCATGCCATCCGTAAGATGCTTTTCTGTGACTGGTGAGTACTCAACCAAGTCATTCTGAGAATAGTGTATGCGGCGACCGAGTTGCTCTTGCCCGGCGTCAATACGGGATAATACCGCGCCACATAGCAGAACTTTAAAAGTGCTCATCATTGGAAAACGTTCTTCGGGGCGAAAACTCTCAAGGATCTTACCGCTGTTGAGATCCAGTTCGATGTAACCCACTCGTGCACCCAACTGATCTTCAGCATCTTTTACTTTCACCAGCGTTTCTGGGTGAGCAAAAACAGGAAGGCAAAATGCCGCAAAAAAGGGAATAAGGGCGACACGGAAATGTTGAATACTCATACTCTTCCTTTTTCAATATTATTGAAGCATTTATCAGGGTTATTGTCTCATGAGCGGATACATATTTGAATGTATTTAGAAAAATAAACAAATAGGGGTTCCGCGCACATTTCCCCGAAAAGTGCCACCTGACGTC

1. **mApple-Myo10 CC mutant**

Size: 12405 bp

cloning sites: 5’ – NotI & 3’ – XbaI

CMV promoter

KOZAK

FLAG-tag

mApple

linker

FL-MyoX+ ***I890A, L893Q, I897A, K904A point mutations***

bGH PA terminator

GACGGATCGGGAGATCTCCCGATCCCCTATGGTGCACTCTCAGTACAATCTGCTCTGATGCCGCATAGTTAAGCCAGTATCTGCTCCCTGCTTGTGTGTTGGAGGTCGCTGAGTAGTGCGCGAGCAAAATTTAAGCTACAACAAGGCAAGGCTTGACCGACAATTGCATGAAGAATCTGCTTAGGGTTAGGCGTTTTGCGCTGCTTCGCGATGTACGGGCCAGATATACGCGTTGACATTGATTATTGACTAGTTATTAATAGTAATCAATTACGGGGTCATTAGTTCATAGCCCATATATGGAGTTCCGCGTTACATAACTTACGGTAAATGGCCCGCCTGGCTGACCGCCCAACGACCCCCGCCCATTGACGTCAATAATGACGTATGTTCCCATAGTAACGCCAATAGGGACTTTCCATTGACGTCAATGGGTGGAGTATTTACGGTAAACTGCCCACTTGGCAGTACATCAAGTGTATCATATGCCAAGTACGCCCCCTATTGACGTCAATGACGGTAAATGGCCCGCCTGGCATTATGCCCAGTACATGACCTTATGGGACTTTCCTACTTGGCAGTACATCTACGTATTAGTCATCGCTATTACCATGGTGATGCGGTTTTGGCAGTACATCAATGGGCGTGGATAGCGGTTTGACTCACGGGGATTTCCAAGTCTCCACCCCATTGACGTCAATGGGAGTTTGTTTTGGCACCAAAATCAACGGGACTTTCCAAAATGTCGTAACAACTCCGCCCCATTGACGCAAATGGGCGGTAGGCGTGTACGGTGGGAGGTCTATATAAGCAGAGCTCTCTGGCTAACTAGAGAACCCACTGCTTACTGGCTTATCGAAATTAATACGACTCACTATAGGGAGACCCAAGCTGGCTAGTTAAGCTTGGTACCGAGCTCGGATCCACTAGTCCAGTGTGGTGGAATTCTGCAGATATCCAGCACAGTGGCGGCCgccaccATGgactacaaggacgatgacgacaagGGCGTGAGCAAGGGCGAGGAGAATAACATGGCCATCATCAAGGAGTTCATGCGCTTCAAGGTGCACATGGAGGGCTCCGTGAACGGCCACGAGTTCGAGATCGAGGGCGAGGGCGAGGGCCGCCCCTACGAGGCCTTTCAGACCGCTAAGCTGAAGGTGACCAAGGGTGGCCCCCTGCCCTTCGCCTGGGACATCCTGTCCCCTCAGTTCATGTACGGCTCCAAGGTCTACATTAAGCACCCAGCCGACATCCCCGACTACTTCAAGCTGTCCTTCCCCGAGGGCTTCAGGTGGGAGCGCGTGATGAACTTCGAGGACGGCGGCATTATTCACGTTAACCAGGACTCCTCCCTGCAGGACGGCGTGTTCATCTACAAGGTGAAGCTGCGCGGCACCAACTTCCCCTCCGACGGCCCCGTAATGCAGAAGAAGACCATGGGCTGGGAGGCCTCCGAGGAGCGGATGTACCCCGAGGACGGCGCCCTGAAGAGCGAGATCAAGAAGAGGCTGAAGCTGAAGGACGGCGGCCACTACGCCGCCGAGGTCAAGACCACCTACAAGGCCAAGAAGCCCGTGCAGCTGCCCGGCGCCTACATCGTCGACATCAAGTTGGACATCGTGTCCCACAACGAGGACTACACCATCGTGGAACAGTACGAACGCGCCGAGGGCCGCCACTCCACCGGCGGCATGGACGAGCTGTACAAGggcggccgaGATAACTTCTTCACCGAGGGAACACGGGTCTGGCTGAGAGAAAATGGCCAGCATTTTCCAAGTACTGTAAATTCCTGTGCAGAAGGCATCGTCGTCTTCCGGACAGACTATGGTCAGGTATTCACTTACAAGCAGAGCACAATTACCCACCAGAAGGTGACTGCTATGCACCCCACGAACGAGGAGGGCGTGGATGACATGGCGTCCTTGACAGAGCTCCATGGCGGCTCCATCATGTATAACTTATTCCAGCGGTATAAGAGAAATCAAATATATACCTACATCGGCTCCATCCTGGCCTCTGTGAACCCCTACCAGCCCATCGCCGGGCTGTACGAGCCTGCCACCATGGAGCAGTACAGCCGGCGCCACCTGGGCGAGCTGCCCCCGCACATCTTCGCCATCGCCAACGAGTGCTACCGCTGCCTGTGGAAGCGCCACGACAACCAGTGCATCCTCATCAGTGGTGAAAGTGGGGCAGGTAAAACCGAAAGCACTAAATTGATCCTCAAGTTTCTGTCAGTCATCAGTCAACAGTCTTTGGAATTGTCCTTAAAGGAGAAGACATCCTGTGTTGAACGAGCTATTCTTGAAAGCAGCCCCATCATGGAAGCTTTCGGCAATGCGAAGACCGTGTACAACAACAACTCTAGTCGCTTTGGGAAGTTTGTTCAGCTGAACATCTGTCAGAAAGGAAATATTCAGGGCGGGAGAATTGTAGATTATTTATTAGAAAAAAACCGAGTAGTAAGGCAAAATCCCGGGGAAAGGAATTATCACATATTTTATGCACTGCTGGCAGGGCTGGAACATGAAGAAAGAGAAGAATTTTATTTATCTACGCCAGAAAACTACCACTACTTGAATCAGTCTGGATGTGTAGAAGACAAGACAATCAGTGACCAGGAATCCTTTAGGGAAGTTATTACGGCAATGGACGTGATGCAGTTCAGCAAGGAGGAAGTTCGGGAAGTGTCGAGGCTGCTTGCTGGTATACTGCATCTTGGGAACATAGAATTTATCACTGCTGGTGGGGCACAGGTTTCCTTCAAAACAGCTTTGGGCAGATCTGCGGAGTTACTTGGGCTGGACCCAACACAGCTCACAGATGCTTTGACCCAGAGATCAATGTTCCTCAGGGGAGAAGAGATCCTCACGCCTCTCAATGTTCAACAGGCAGTAGACAGCAGGGACTCCCTGGCCATGGCTCTGTATGCGTGCTGCTTTGAGTGGGTAATCAAGAAGATCAACAGCAGGATCAAAGGCAATGAGGACTTCAAGTCTATTGGCATCCTCGACATCTTTGGATTTGAAAACTTTGAGGTTAATCACTTTGAACAGTTCAATATAAACTATGCAAACGAGAAACTTCAGGAGTACTTCAACAAGCATATTTTTTCTTTAGAACAACTAGAATATAGCCGGGAAGGATTAGTGTGGGAAGATATTGACTGGATAGACAATGGAGAATGCCTGGACTTGATTGAGAAGAAACTTGGCCTCCTAGCCCTTATCAATGAAGAAAGCCATTTTCCTCAAGCCACAGACAGCACCTTATTGGAGAAGCTACACAGTCAGCATGCGAATAACCACTTTTATGTGAAGCCCAGAGTTGCAGTTAACAATTTTGGAGTGAAGCACTATGCTGGAGAGGTGCAATATGATGTCCGAGGTATCTTGGAGAAGAACAGAGATACATTTCGAGATGACCTTCTCAATTTGCTAAGAGAAAGCCGATTTGACTTTATCTACGATCTTTTTGAACATGTTTCAAGCCGCAACAACCAGGATACCTTGAAATGTGGAAGCAAACATCGGCGGCCTACAGTCAGCTCACAGTTCAAGGACTCACTGCATTCCTTAATGGCAACGCTAAGCTCCTCTAATCCTTTCTTTGTTCGCTGTATCAAGCCAAACATGCAGAAGATGCCAGACCAGTTTGACCAGGCGGTTGTGCTGAACCAGCTGCGGTACTCAGGGATGCTGGAGACTGTGAGAATCCGCAAAGCTGGGTATGCGGTCCGAAGACCCTTTCAGGACTTTTACAAAAGGTATAAAGTGCTGATGAGGAATCTGGCTCTGCCTGAGGACGTCCGAGGGAAGTGCACGAGCCTGCTGCAGCTCTATGATGCCTCCAACAGCGAGTGGCAGCTGGGGAAGACCAAGGTCTTTCTTCGAGAATCCTTGGAACAGAAACTGGAGAAGCGGAGGGAAGAGGAAGTGAGCCACGCGGCCATGGTGATTCGGGCCCATGTCTTGGGCTTCTTAGCACGAAAACAATACAGAAAGGTCCTTTATTGTGTGGTGATAATACAGAAGAATTACAGAGCATTCCTTCTGAGGAGGAGATTTTTGCACCTGAAAAAGGCAGCCATAGTTTTCCAGAAGCAACTCAGAGGTCAGATTGCTCGGAGAGTTTACAGACAATTGCTGGCAGAGAAAAGGGAGCAAGAAGAAAAGAAGAAACAGGAAGAGGAAGAAAAGAAGAAACGGGAGGAAGAAGAAAGAGAAAGAGAGAGAGAGCGAAGAGAAGCCGAGCTCCGCGCCCAGCAGGAAGAAGAAACGAGGAAGCAGCAAGAACTCGAAGCCTTGCAGAAGAGCCAGAAGGAAGCTGAACTGACCCGTGAACTGGAGAAACAGAAGGAAAATAAGCAGGTGGAAGAG***gcC***CTCCGT***CaG***GAGAAAGAA***gcC***GAGGACCTGCAGCGCATG***gcc***GAGCAGCAGGAGCTGTCGCTGACCGAGGCTTCCCTGCAGAAGCTGCAGGAGCGGCGGGACCAGGAGCTCCGCAGGCTGGAGGAGGAAGCGTGCAGGGCGGCCCAGGAGTTCCTCGAGTCCCTCAATTTCGACGAGATCGACGAGTGTGTCCGGAATATCGAGCGGTCCCTGTCGGTGGGAAGCGAATTTTCCAGCGAGCTGGCTGAGAGCGCATGCGAGGAGAAGCCCAACTTCAACTTCAGCCAGCCCTACCCAGAGGAGGAGGTCGATGAGGGCTTCGAAGCCGACGACGACGCCTTCAAGGACTCCCCCAACCCCAGCGAGCACGGCCACTCAGACCAGCGAACAAGTGGCATCCGGACCAGCGATGACTCTTCAGAGGAGGACCCATACATGAACGACACGGTGGTGCCCACCAGCCCCAGTGCGGACAGCACGGTGCTGCTCGCCCCATCAGTGCAGGACTCCGGGAGCCTACACAACTCCTCCAGCGGCGAGTCCACCTACTGCATGCCCCAGAACGCTGGGGACTTGCCCTCCCCAGACGGCGACTACGACTACGACCAGGATGACTATGAGGACGGTGCCATCACTTCCGGCAGCAGCGTGACCTTCTCCAACTCCTACGGCAGCCAGTGGTCCCCCGACTACCGCTGCTCTGTGGGGACCTACAACAGCTCGGGTGCCTACCGGTTCAGCTCTGAGGGGGCGCAGTCCTCGTTTGAAGATAGTGAAGAGGACTTTGATTCCAGGTTTGATACAGATGATGAGCTTTCATACCGGCGTGACTCTGTGTACAGCTGTGTCACTCTGCCGTATTTCCACAGCTTTCTGTACATGAAAGGTGGCCTGATGAACTCTTGGAAACGCCGCTGGTGCGTCCTCAAGGATGAAACCTTCTTGTGGTTCCGCTCCAAGCAGGAGGCCCTCAAGCAAGGCTGGCTCCACAAAAAAGGGGGGGGCTCCTCCACGCTGTCCAGGAGAAATTGGAAGAAGCGCTGGTTTGTCCTCCGCCAGTCCAAGCTGATGTACTTTGAAAACGACAGCGAGGAGAAGCTCAAGGGCACCGTAGAAGTGCGAACGGCAAAAGAGATCATAGATAACACCACCAAGGAGAATGGGATCGACATCATTATGGCCGATAGGACTTTCCACCTGATTGCAGAGTCCCCAGAAGATGCCAGCCAGTGGTTCAGCGTGCTGAGTCAGGTCCACGCGTCCACGGACCAGGAGATCCAGGAGATGCATGATGAGCAGGCAAACCCACAGAATGCTGTGGGCACCTTGGATGTGGGGCTGATTGATTCTGTGTGTGCCTCTGACAGCCCTGATAGACCCAACTCGTTTGTGATCATCACGGCCAACCGGGTGCTGCACTGCAACGCCGACACGCCGGAGGAGATGCACCACTGGATAACCCTGCTGCAGAGGTCCAAAGGGGACACCAGAGTGGAGGGCCAGGAATTCATCGTGAGAGGATGGTTGCACAAAGAGGTGAAGAACAGTCCAAAGATGTCTTCACTGAAACTGAAGAAACGGTGGTTTGTACTCACCCACAATTCCCTGGATTACTACAAGAGTTCAGAGAAGAACGCGCTCAAACTGGGGACCCTGGTCCTCAACAGCCTCTGCTCTGTCGTCCCCCCAGATGAGAAGATATTCAAAGAGACAGGCTACTGGAACGTCACCGTGTACGGGCGCAAGCACTGTTACCGGCTCTACACCAAGCTGCTCAACGAGGCCACCCGGTGGTCCAGTGCCATTCAAAACGTGACTGACACCAAGGCCCCGATCGACACCCCCACCCAGCAGCTGATTCAAGATATCAAGGAGAACTGCCTGAACTCGGATGTGGTGGAACAGATTTACAAGCGGAACCCGATCCTTCGATACACCCATCACCCCTTGCACTCCCCGCTCCTGCCCCTTCCGTATGGGGACATAAATCTCAACTTGCTCAAAGACAAAGGCTATACCACCCTTCAGGATGAGGCCATCAAGATATTCAATTCCCTGCAGCAACTGGAGTCCATGTCTGACCCAATTCCAATAATCCAGGGCATCCTACAGACAGGGCATGACCTGCGACCTCTGCGGGACGAGCTGTACTGCCAGCTTATCAAACAGACCAACAAAGTGCCCCACCCCGGCAGTGTGGGCAACCTGTACAGCTGGCAGATCCTGACATGCCTGAGCTGCACCTTCCTGCCGAGTCGAGGGATTCTCAAGTATCTCAAGTTCCATCTGAAAAGGATACGGGAACAGTTTCCAGGAACCGAGATGGAAAAATACGCTCTCTTCACTTACGAATCTCTTAAGAAAACCAAATGCCGAGAGTTTGTGCCTTCCCGAGATGAAATAGAAGCTCTGATCCACAGGCAGGAAATGACATCCACGGTCTATTGCCATGGCGGCGGCTCCTGCAAGATCACCATCAACTCCCACACCACCGCTGGGGAGGTGGTGGAGAAGCTGATCCGAGGCCTGGCCATGGAGGACAGCAGGAACATGTTTGCTTTGTTTGAATACAACGGCCACGTCGACAAAGCCATTGAAAGTCGAACCGTCGTAGCTGATGTCTTAGCCAAGTTTGAAAAGCTGGCTGCCACATCCGAGGTTGGGGACCTGCCATGGAAATTCTACTTCAAACTTTACTGCTTCCTGGACACAGACAACGTGCCAAAAGACAGTGTGGAGTTTGCATTTATGTTTGAACAGGCCCACGAAGCGGTTATCCATGGCCACCATCCAGCCCCGGAAGAAAACCTCCAGGTTCTTGCTGCCCTGCGACTCCAGTATCTGCAGGGGGATTATACTCTGCACGCTGCCATCCCACCTCTCGAAGAGGTTTATTCCCTGCAGAGACTCAAGGCCCGCATCAGCCAGTCAACCAAAACCTTCACCCCTTGTGAACGGCTGGAGAAGAGGCGGACGAGCTTCCTAGAGGGGACCCTGAGGCGGAGCTTCCGGACAGGATCCGTGGTCCGGCAGAAGGTCGAGGAGGAGCAGATGCTGGACATGTGGATTAAGGAAGAAGTCTCCTCTGCTCGAGCCAGTATCATTGACAAGTGGAGGAAATTTCAGGGAATGAACCAGGAACAGGCCATGGCCAAGTACATGGCCTTGATCAAGGAGTGGCCTGGCTATGGCTCGACGCTGTTTGATGTGGAGTGCAAGGAAGGTGGCTTCCCTCAGGAACTCTGGTTGGGTGTCAGCGCGGACGCCGTCTCCGTCTACAAGCGTGGAGAGGGAAGACCACTGGAAGTCTTCCAGTATGAACACATCCTCTCTTTTGGGGCACCCCTGGCGAATACGTATAAGATCGTGGTCGATGAGAGGGAGCTGCTCTTTGAAACCAGTGAGGTGGTGGATGTGGCCAAGCTCATGAAAGCCTACATCAGCATGATCGTGAAGAAGCGCTACAGCACGACACGCTCCGCCAGCAGCCAGGGCAGCTCCAGGTAATCTAGAGGGCCCTTCGAACAAAAACTCATCTCAGAAGAGGATCTGAATATGCATACCGGTCATCATCACCATCACCATTGAGTTTAAACCCGCTGATCAGCCTCGACTGTGCCTTCTAGTTGCCAGCCATCTGTTGTTTGCCCCTCCCCCGTGCCTTCCTTGACCCTGGAAGGTGCCACTCCCACTGTCCTTTCCTAATAAAATGAGGAAATTGCATCGCATTGTCTGAGTAGGTGTCATTCTATTCTGGGGGGTGGGGTGGGGCAGGACAGCAAGGGGGAGGATTGGGAAGACAATAGCAGGCATGCTGGGGATGCGGTGGGCTCTATGGCTTCTGAGGCGGAAAGAACCAGCTGGGGCTCTAGGGGGTATCCCCACGCGCCCTGTAGCGGCGCATTAAGCGCGGCGGGTGTGGTGGTTACGCGCAGCGTGACCGCTACACTTGCCAGCGCCCTAGCGCCCGCTCCTTTCGCTTTCTTCCCTTCCTTTCTCGCCACGTTCGCCGGCTTTCCCCGTCAAGCTCTAAATCGGGGGCTCCCTTTAGGGTTCCGATTTAGTGCTTTACGGCACCTCGACCCCAAAAAACTTGATTAGGGTGATGGTTCACGTAGTGGGCCATCGCCCTGATAGACGGTTTTTCGCCCTTTGACGTTGGAGTCCACGTTCTTTAATAGTGGACTCTTGTTCCAAACTGGAACAACACTCAACCCTATCTCGGTCTATTCTTTTGATTTATAAGGGATTTTGCCGATTTCGGCCTATTGGTTAAAAAATGAGCTGATTTAACAAAAATTTAACGCGAATTAATTCTGTGGAATGTGTGTCAGTTAGGGTGTGGAAAGTCCCCAGGCTCCCCAGCAGGCAGAAGTATGCAAAGCATGCATCTCAATTAGTCAGCAACCAGGTGTGGAAAGTCCCCAGGCTCCCCAGCAGGCAGAAGTATGCAAAGCATGCATCTCAATTAGTCAGCAACCATAGTCCCGCCCCTAACTCCGCCCATCCCGCCCCTAACTCCGCCCAGTTCCGCCCATTCTCCGCCCCATGGCTGACTAATTTTTTTTATTTATGCAGAGGCCGAGGCCGCCTCTGCCTCTGAGCTATTCCAGAAGTAGTGAGGAGGCTTTTTTGGAGGCCTAGGCTTTTGCAAAAAGCTCCCGGGAGCTTGTATATCCATTTTCGGATCTGATCAAGAGACAGGATGAGGATCGTTTCGCATGATTGAACAAGATGGATTGCACGCAGGTTCTCCGGCCGCTTGGGTGGAGAGGCTATTCGGCTATGACTGGGCACAACAGACAATCGGCTGCTCTGATGCCGCCGTGTTCCGGCTGTCAGCGCAGGGGCGCCCGGTTCTTTTTGTCAAGACCGACCTGTCCGGTGCCCTGAATGAACTGCAGGACGAGGCAGCGCGGCTATCGTGGCTGGCCACGACGGGCGTTCCTTGCGCAGCTGTGCTCGACGTTGTCACTGAAGCGGGAAGGGACTGGCTGCTATTGGGCGAAGTGCCGGGGCAGGATCTCCTGTCATCTCACCTTGCTCCTGCCGAGAAAGTATCCATCATGGCTGATGCAATGCGGCGGCTGCATACGCTTGATCCGGCTACCTGCCCATTCGACCACCAAGCGAAACATCGCATCGAGCGAGCACGTACTCGGATGGAAGCCGGTCTTGTCGATCAGGATGATCTGGACGAAGAGCATCAGGGGCTCGCGCCAGCCGAACTGTTCGCCAGGCTCAAGGCGCGCATGCCCGACGGCGAGGATCTCGTCGTGACCCATGGCGATGCCTGCTTGCCGAATATCATGGTGGAAAATGGCCGCTTTTCTGGATTCATCGACTGTGGCCGGCTGGGTGTGGCGGACCGCTATCAGGACATAGCGTTGGCTACCCGTGATATTGCTGAAGAGCTTGGCGGCGAATGGGCTGACCGCTTCCTCGTGCTTTACGGTATCGCCGCTCCCGATTCGCAGCGCATCGCCTTCTATCGCCTTCTTGACGAGTTCTTCTGAGCGGGACTCTGGGGTTCGAAATGACCGACCAAGCGACGCCCAACCTGCCATCACGAGATTTCGATTCCACCGCCGCCTTCTATGAAAGGTTGGGCTTCGGAATCGTTTTCCGGGACGCCGGCTGGATGATCCTCCAGCGCGGGGATCTCATGCTGGAGTTCTTCGCCCACCCCAACTTGTTTATTGCAGCTTATAATGGTTACAAATAAAGCAATAGCATCACAAATTTCACAAATAAAGCATTTTTTTCACTGCATTCTAGTTGTGGTTTGTCCAAACTCATCAATGTATCTTATCATGTCTGTATACCGTCGACCTCTAGCTAGAGCTTGGCGTAATCATGGTCATAGCTGTTTCCTGTGTGAAATTGTTATCCGCTCACAATTCCACACAACATACGAGCCGGAAGCATAAAGTGTAAAGCCTGGGGTGCCTAATGAGTGAGCTAACTCACATTAATTGCGTTGCGCTCACTGCCCGCTTTCCAGTCGGGAAACCTGTCGTGCCAGCTGCATTAATGAATCGGCCAACGCGCGGGGAGAGGCGGTTTGCGTATTGGGCGCTCTTCCGCTTCCTCGCTCACTGACTCGCTGCGCTCGGTCGTTCGGCTGCGGCGAGCGGTATCAGCTCACTCAAAGGCGGTAATACGGTTATCCACAGAATCAGGGGATAACGCAGGAAAGAACATGTGAGCAAAAGGCCAGCAAAAGGCCAGGAACCGTAAAAAGGCCGCGTTGCTGGCGTTTTTCCATAGGCTCCGCCCCCCTGACGAGCATCACAAAAATCGACGCTCAAGTCAGAGGTGGCGAAACCCGACAGGACTATAAAGATACCAGGCGTTTCCCCCTGGAAGCTCCCTCGTGCGCTCTCCTGTTCCGACCCTGCCGCTTACCGGATACCTGTCCGCCTTTCTCCCTTCGGGAAGCGTGGCGCTTTCTCATAGCTCACGCTGTAGGTATCTCAGTTCGGTGTAGGTCGTTCGCTCCAAGCTGGGCTGTGTGCACGAACCCCCCGTTCAGCCCGACCGCTGCGCCTTATCCGGTAACTATCGTCTTGAGTCCAACCCGGTAAGACACGACTTATCGCCACTGGCAGCAGCCACTGGTAACAGGATTAGCAGAGCGAGGTATGTAGGCGGTGCTACAGAGTTCTTGAAGTGGTGGCCTAACTACGGCTACACTAGAAGAACAGTATTTGGTATCTGCGCTCTGCTGAAGCCAGTTACCTTCGGAAAAAGAGTTGGTAGCTCTTGATCCGGCAAACAAACCACCGCTGGTAGCGGTTTTTTTGTTTGCAAGCAGCAGATTACGCGCAGAAAAAAAGGATCTCAAGAAGATCCTTTGATCTTTTCTACGGGGTCTGACGCTCAGTGGAACGAAAACTCACGTTAAGGGATTTTGGTCATGAGATTATCAAAAAGGATCTTCACCTAGATCCTTTTAAATTAAAAATGAAGTTTTAAATCAATCTAAAGTATATATGAGTAAACTTGGTCTGACAGTTACCAATGCTTAATCAGTGAGGCACCTATCTCAGCGATCTGTCTATTTCGTTCATCCATAGTTGCCTGACTCCCCGTCGTGTAGATAACTACGATACGGGAGGGCTTACCATCTGGCCCCAGTGCTGCAATGATACCGCGAGACCCACGCTCACCGGCTCCAGATTTATCAGCAATAAACCAGCCAGCCGGAAGGGCCGAGCGCAGAAGTGGTCCTGCAACTTTATCCGCCTCCATCCAGTCTATTAATTGTTGCCGGGAAGCTAGAGTAAGTAGTTCGCCAGTTAATAGTTTGCGCAACGTTGTTGCCATTGCTACAGGCATCGTGGTGTCACGCTCGTCGTTTGGTATGGCTTCATTCAGCTCCGGTTCCCAACGATCAAGGCGAGTTACATGATCCCCCATGTTGTGCAAAAAAGCGGTTAGCTCCTTCGGTCCTCCGATCGTTGTCAGAAGTAAGTTGGCCGCAGTGTTATCACTCATGGTTATGGCAGCACTGCATAATTCTCTTACTGTCATGCCATCCGTAAGATGCTTTTCTGTGACTGGTGAGTACTCAACCAAGTCATTCTGAGAATAGTGTATGCGGCGACCGAGTTGCTCTTGCCCGGCGTCAATACGGGATAATACCGCGCCACATAGCAGAACTTTAAAAGTGCTCATCATTGGAAAACGTTCTTCGGGGCGAAAACTCTCAAGGATCTTACCGCTGTTGAGATCCAGTTCGATGTAACCCACTCGTGCACCCAACTGATCTTCAGCATCTTTTACTTTCACCAGCGTTTCTGGGTGAGCAAAAACAGGAAGGCAAAATGCCGCAAAAAAGGGAATAAGGGCGACACGGAAATGTTGAATACTCATACTCTTCCTTTTTCAATATTATTGAAGCATTTATCAGGGTTATTGTCTCATGAGCGGATACATATTTGAATGTATTTAGAAAAATAAACAAATAGGGGTTCCGCGCACATTTCCCCGAAAAGTGCCACCTGACGTC

1. **mApple-Myo10 HMM**

size: 9225 bp

cloning sites: 5’ – NotI & 3’ – XbaI

CMV promoter

KOZAK

FLAG-tag

mApple

linker

Hum MyoX (A938)

Leucine zipper

bGH PA terminator

GACGGATCGGGAGATCTCCCGATCCCCTATGGTGCACTCTCAGTACAATCTGCTCTGATGCCGCATAGTTAAGCCAGTATCTGCTCCCTGCTTGTGTGTTGGAGGTCGCTGAGTAGTGCGCGAGCAAAATTTAAGCTACAACAAGGCAAGGCTTGACCGACAATTGCATGAAGAATCTGCTTAGGGTTAGGCGTTTTGCGCTGCTTCGCGATGTACGGGCCAGATATACGCGTTGACATTGATTATTGACTAGTTATTAATAGTAATCAATTACGGGGTCATTAGTTCATAGCCCATATATGGAGTTCCGCGTTACATAACTTACGGTAAATGGCCCGCCTGGCTGACCGCCCAACGACCCCCGCCCATTGACGTCAATAATGACGTATGTTCCCATAGTAACGCCAATAGGGACTTTCCATTGACGTCAATGGGTGGAGTATTTACGGTAAACTGCCCACTTGGCAGTACATCAAGTGTATCATATGCCAAGTACGCCCCCTATTGACGTCAATGACGGTAAATGGCCCGCCTGGCATTATGCCCAGTACATGACCTTATGGGACTTTCCTACTTGGCAGTACATCTACGTATTAGTCATCGCTATTACCATGGTGATGCGGTTTTGGCAGTACATCAATGGGCGTGGATAGCGGTTTGACTCACGGGGATTTCCAAGTCTCCACCCCATTGACGTCAATGGGAGTTTGTTTTGGCACCAAAATCAACGGGACTTTCCAAAATGTCGTAACAACTCCGCCCCATTGACGCAAATGGGCGGTAGGCGTGTACGGTGGGAGGTCTATATAAGCAGAGCTCTCTGGCTAACTAGAGAACCCACTGCTTACTGGCTTATCGAAATTAATACGACTCACTATAGGGAGACCCAAGCTGGCTAGTTAAGCTTGGTACCGAGCTCGGATCCACTAGTCCAGTGTGGTGGAATTCTGCAGATATCCAGCACAGTGGCGGCCgccaccATGgactacaaggacgatgacgacaagGGCGTGAGCAAGGGCGAGGAGAATAACATGGCCATCATCAAGGAGTTCATGCGCTTCAAGGTGCACATGGAGGGCTCCGTGAACGGCCACGAGTTCGAGATCGAGGGCGAGGGCGAGGGCCGCCCCTACGAGGCCTTTCAGACCGCTAAGCTGAAGGTGACCAAGGGTGGCCCCCTGCCCTTCGCCTGGGACATCCTGTCCCCTCAGTTCATGTACGGCTCCAAGGTCTACATTAAGCACCCAGCCGACATCCCCGACTACTTCAAGCTGTCCTTCCCCGAGGGCTTCAGGTGGGAGCGCGTGATGAACTTCGAGGACGGCGGCATTATTCACGTTAACCAGGACTCCTCCCTGCAGGACGGCGTGTTCATCTACAAGGTGAAGCTGCGCGGCACCAACTTCCCCTCCGACGGCCCCGTAATGCAGAAGAAGACCATGGGCTGGGAGGCCTCCGAGGAGCGGATGTACCCCGAGGACGGCGCCCTGAAGAGCGAGATCAAGAAGAGGCTGAAGCTGAAGGACGGCGGCCACTACGCCGCCGAGGTCAAGACCACCTACAAGGCCAAGAAGCCCGTGCAGCTGCCCGGCGCCTACATCGTCGACATCAAGTTGGACATCGTGTCCCACAACGAGGACTACACCATCGTGGAACAGTACGAACGCGCCGAGGGCCGCCACTCCACCGGCGGCATGGACGAGCTGTACAAGggcggccgaATGGATAACTTCTTCACCGAGGGAACACGGGTCTGGCTGAGAGAAAATGGCCAGCATTTTCCAAGTACTGTAAATTCCTGTGCAGAAGGCATCGTCGTCTTCCGGACAGACTATGGTCAGGTATTCACTTACAAGCAGAGCACAATTACCCACCAGAAGGTGACTGCTATGCACCCCACGAACGAGGAGGGCGTGGATGACATGGCGTCCTTGACAGAGCTCCATGGCGGCTCCATCATGTATAACTTATTCCAGCGGTATAAGAGAAATCAAATATATACCTACATCGGCTCCATCCTGGCCTCTGTGAACCCCTACCAGCCCATCGCCGGGCTGTACGAGCCTGCCACCATGGAGCAGTACAGCCGGCGCCACCTGGGCGAGCTGCCCCCGCACATCTTCGCCATCGCCAACGAGTGCTACCGCTGCCTGTGGAAGCGCCACGACAACCAGTGCATCCTCATCAGTGGTGAAAGTGGGGCAGGTAAAACCGAAAGCACTAAATTGATCCTCAAGTTTCTGTCAGTCATCAGTCAACAGTCTTTGGAATTGTCCTTAAAGGAGAAGACATCCTGTGTTGAACGAGCTATTCTTGAAAGCAGCCCCATCATGGAAGCTTTCGGCAATGCGAAGACCGTGTACAACAACAACTCTAGTCGCTTTGGGAAGTTTGTTCAGCTGAACATCTGTCAGAAAGGAAATATTCAGGGCGGGAGAATTGTAGATTATTTATTAGAAAAAAACCGAGTAGTAAGGCAAAATCCCGGGGAAAGGAATTATCACATATTTTATGCACTGCTGGCAGGGCTGGAACATGAAGAAAGAGAAGAATTTTATTTATCTACGCCAGAAAACTACCACTACTTGAATCAGTCTGGATGTGTAGAAGACAAGACAATCAGTGACCAGGAATCCTTTAGGGAAGTTATTACGGCAATGGACGTGATGCAGTTCAGCAAGGAGGAAGTTCGGGAAGTGTCGAGGCTGCTTGCTGGTATACTGCATCTTGGGAACATAGAATTTATCACTGCTGGTGGGGCACAGGTTTCCTTCAAAACAGCTTTGGGCAGATCTGCGGAGTTACTTGGGCTGGACCCAACACAGCTCACAGATGCTTTGACCCAGAGATCAATGTTCCTCAGGGGAGAAGAGATCCTCACGCCTCTCAATGTTCAACAGGCAGTAGACAGCAGGGACTCCCTGGCCATGGCTCTGTATGCGTGCTGCTTTGAGTGGGTAATCAAGAAGATCAACAGCAGGATCAAAGGCAATGAGGACTTCAAGTCTATTGGCATCCTCGACATCTTTGGATTTGAAAACTTTGAGGTTAATCACTTTGAACAGTTCAATATAAACTATGCAAACGAGAAACTTCAGGAGTACTTCAACAAGCATATTTTTTCTTTAGAACAACTAGAATATAGCCGGGAAGGATTAGTGTGGGAAGATATTGACTGGATAGACAATGGAGAATGCCTGGACTTGATTGAGAAGAAACTTGGCCTCCTAGCCCTTATCAATGAAGAAAGCCATTTTCCTCAAGCCACAGACAGCACCTTATTGGAGAAGCTACACAGTCAGCATGCGAATAACCACTTTTATGTGAAGCCCAGAGTTGCAGTTAACAATTTTGGAGTGAAGCACTATGCTGGAGAGGTGCAATATGATGTCCGAGGTATCTTGGAGAAGAACAGAGATACATTTCGAGATGACCTTCTCAATTTGCTAAGAGAAAGCCGATTTGACTTTATCTACGATCTTTTTGAACATGTTTCAAGCCGCAACAACCAGGATACCTTGAAATGTGGAAGCAAACATCGGCGGCCTACAGTCAGCTCACAGTTCAAGGACTCACTGCATTCCTTAATGGCAACGCTAAGCTCCTCTAATCCTTTCTTTGTTCGCTGTATCAAGCCAAACATGCAGAAGATGCCAGACCAGTTTGACCAGGCGGTTGTGCTGAACCAGCTGCGGTACTCAGGGATGCTGGAGACTGTGAGAATCCGCAAAGCTGGGTATGCGGTCCGAAGACCCTTTCAGGACTTTTACAAAAGGTATAAAGTGCTGATGAGGAATCTGGCTCTGCCTGAGGACGTCCGAGGGAAGTGCACGAGCCTGCTGCAGCTCTATGATGCCTCCAACAGCGAGTGGCAGCTGGGGAAGACCAAGGTCTTTCTTCGAGAATCCTTGGAACAGAAACTGGAGAAGCGGAGGGAAGAGGAAGTGAGCCACGCGGCCATGGTGATTCGGGCCCATGTCTTGGGCTTCTTAGCACGAAAACAATACAGAAAGGTCCTTTATTGTGTGGTGATAATACAGAAGAATTACAGAGCATTCCTTCTGAGGAGGAGATTTTTGCACCTGAAAAAGGCAGCCATAGTTTTCCAGAAGCAACTCAGAGGTCAGATTGCTCGGAGAGTTTACAGACAATTGCTGGCAGAGAAAAGGGAGCAAGAAGAAAAGAAGAAACAGGAAGAGGAAGAAAAGAAGAAACGGGAGGAAGAAGAAAGAGAAAGAGAGAGAGAGCGAAGAGAAGCCGAGCTCCGCGCCCAGCAGGAAGAAGAAACGAGGAAGCAGCAAGAACTCGAAGCCTTGCAGAAGAGCCAGAAGGAAGCTGAACTGACCCGTGAACTGGAGAAACAGAAGGAAAATAAGCAGGTGGAAGAGATCCTCCGTCTGGAGAAAGAAATCGAGGACCTGCAGCGCATGAAGGAGCAGCAGGAGCTGTCGCTGACCGAGGCTTCCCTGCAGAAGCTGCAGGAGCGGCGGGACCAGGAGCTCCGCAGGCTCGAGGAGGAAGCGTGCAGGGCGGCCtccgagggaggaagcggcggcagcggcggcagcggcggcagcgcggcgagcgcggcgATGAAACAGCTAGAGGACAAAGTAGAGGAGCTGCTGTCCAAGAATTACCATCTGGAAAATGAGGTCGCGAGACTAAAGAAGCTAGTCGGGGAGggcggcgactacaaggacgatgacgacaagTAGTCTAGAGGGCCCTTCGAACAAAAACTCATCTCAGAAGAGGATCTGAATATGCATACCGGTCATCATCACCATCACCATTGAGTTTAAACCCGCTGATCAGCCTCGACTGTGCCTTCTAGTTGCCAGCCATCTGTTGTTTGCCCCTCCCCCGTGCCTTCCTTGACCCTGGAAGGTGCCACTCCCACTGTCCTTTCCTAATAAAATGAGGAAATTGCATCGCATTGTCTGAGTAGGTGTCATTCTATTCTGGGGGGTGGGGTGGGGCAGGACAGCAAGGGGGAGGATTGGGAAGACAATAGCAGGCATGCTGGGGATGCGGTGGGCTCTATGGCTTCTGAGGCGGAAAGAACCAGCTGGGGCTCTAGGGGGTATCCCCACGCGCCCTGTAGCGGCGCATTAAGCGCGGCGGGTGTGGTGGTTACGCGCAGCGTGACCGCTACACTTGCCAGCGCCCTAGCGCCCGCTCCTTTCGCTTTCTTCCCTTCCTTTCTCGCCACGTTCGCCGGCTTTCCCCGTCAAGCTCTAAATCGGGGGCTCCCTTTAGGGTTCCGATTTAGTGCTTTACGGCACCTCGACCCCAAAAAACTTGATTAGGGTGATGGTTCACGTAGTGGGCCATCGCCCTGATAGACGGTTTTTCGCCCTTTGACGTTGGAGTCCACGTTCTTTAATAGTGGACTCTTGTTCCAAACTGGAACAACACTCAACCCTATCTCGGTCTATTCTTTTGATTTATAAGGGATTTTGCCGATTTCGGCCTATTGGTTAAAAAATGAGCTGATTTAACAAAAATTTAACGCGAATTAATTCTGTGGAATGTGTGTCAGTTAGGGTGTGGAAAGTCCCCAGGCTCCCCAGCAGGCAGAAGTATGCAAAGCATGCATCTCAATTAGTCAGCAACCAGGTGTGGAAAGTCCCCAGGCTCCCCAGCAGGCAGAAGTATGCAAAGCATGCATCTCAATTAGTCAGCAACCATAGTCCCGCCCCTAACTCCGCCCATCCCGCCCCTAACTCCGCCCAGTTCCGCCCATTCTCCGCCCCATGGCTGACTAATTTTTTTTATTTATGCAGAGGCCGAGGCCGCCTCTGCCTCTGAGCTATTCCAGAAGTAGTGAGGAGGCTTTTTTGGAGGCCTAGGCTTTTGCAAAAAGCTCCCGGGAGCTTGTATATCCATTTTCGGATCTGATCAAGAGACAGGATGAGGATCGTTTCGCATGATTGAACAAGATGGATTGCACGCAGGTTCTCCGGCCGCTTGGGTGGAGAGGCTATTCGGCTATGACTGGGCACAACAGACAATCGGCTGCTCTGATGCCGCCGTGTTCCGGCTGTCAGCGCAGGGGCGCCCGGTTCTTTTTGTCAAGACCGACCTGTCCGGTGCCCTGAATGAACTGCAGGACGAGGCAGCGCGGCTATCGTGGCTGGCCACGACGGGCGTTCCTTGCGCAGCTGTGCTCGACGTTGTCACTGAAGCGGGAAGGGACTGGCTGCTATTGGGCGAAGTGCCGGGGCAGGATCTCCTGTCATCTCACCTTGCTCCTGCCGAGAAAGTATCCATCATGGCTGATGCAATGCGGCGGCTGCATACGCTTGATCCGGCTACCTGCCCATTCGACCACCAAGCGAAACATCGCATCGAGCGAGCACGTACTCGGATGGAAGCCGGTCTTGTCGATCAGGATGATCTGGACGAAGAGCATCAGGGGCTCGCGCCAGCCGAACTGTTCGCCAGGCTCAAGGCGCGCATGCCCGACGGCGAGGATCTCGTCGTGACCCATGGCGATGCCTGCTTGCCGAATATCATGGTGGAAAATGGCCGCTTTTCTGGATTCATCGACTGTGGCCGGCTGGGTGTGGCGGACCGCTATCAGGACATAGCGTTGGCTACCCGTGATATTGCTGAAGAGCTTGGCGGCGAATGGGCTGACCGCTTCCTCGTGCTTTACGGTATCGCCGCTCCCGATTCGCAGCGCATCGCCTTCTATCGCCTTCTTGACGAGTTCTTCTGAGCGGGACTCTGGGGTTCGAAATGACCGACCAAGCGACGCCCAACCTGCCATCACGAGATTTCGATTCCACCGCCGCCTTCTATGAAAGGTTGGGCTTCGGAATCGTTTTCCGGGACGCCGGCTGGATGATCCTCCAGCGCGGGGATCTCATGCTGGAGTTCTTCGCCCACCCCAACTTGTTTATTGCAGCTTATAATGGTTACAAATAAAGCAATAGCATCACAAATTTCACAAATAAAGCATTTTTTTCACTGCATTCTAGTTGTGGTTTGTCCAAACTCATCAATGTATCTTATCATGTCTGTATACCGTCGACCTCTAGCTAGAGCTTGGCGTAATCATGGTCATAGCTGTTTCCTGTGTGAAATTGTTATCCGCTCACAATTCCACACAACATACGAGCCGGAAGCATAAAGTGTAAAGCCTGGGGTGCCTAATGAGTGAGCTAACTCACATTAATTGCGTTGCGCTCACTGCCCGCTTTCCAGTCGGGAAACCTGTCGTGCCAGCTGCATTAATGAATCGGCCAACGCGCGGGGAGAGGCGGTTTGCGTATTGGGCGCTCTTCCGCTTCCTCGCTCACTGACTCGCTGCGCTCGGTCGTTCGGCTGCGGCGAGCGGTATCAGCTCACTCAAAGGCGGTAATACGGTTATCCACAGAATCAGGGGATAACGCAGGAAAGAACATGTGAGCAAAAGGCCAGCAAAAGGCCAGGAACCGTAAAAAGGCCGCGTTGCTGGCGTTTTTCCATAGGCTCCGCCCCCCTGACGAGCATCACAAAAATCGACGCTCAAGTCAGAGGTGGCGAAACCCGACAGGACTATAAAGATACCAGGCGTTTCCCCCTGGAAGCTCCCTCGTGCGCTCTCCTGTTCCGACCCTGCCGCTTACCGGATACCTGTCCGCCTTTCTCCCTTCGGGAAGCGTGGCGCTTTCTCATAGCTCACGCTGTAGGTATCTCAGTTCGGTGTAGGTCGTTCGCTCCAAGCTGGGCTGTGTGCACGAACCCCCCGTTCAGCCCGACCGCTGCGCCTTATCCGGTAACTATCGTCTTGAGTCCAACCCGGTAAGACACGACTTATCGCCACTGGCAGCAGCCACTGGTAACAGGATTAGCAGAGCGAGGTATGTAGGCGGTGCTACAGAGTTCTTGAAGTGGTGGCCTAACTACGGCTACACTAGAAGAACAGTATTTGGTATCTGCGCTCTGCTGAAGCCAGTTACCTTCGGAAAAAGAGTTGGTAGCTCTTGATCCGGCAAACAAACCACCGCTGGTAGCGGTTTTTTTGTTTGCAAGCAGCAGATTACGCGCAGAAAAAAAGGATCTCAAGAAGATCCTTTGATCTTTTCTACGGGGTCTGACGCTCAGTGGAACGAAAACTCACGTTAAGGGATTTTGGTCATGAGATTATCAAAAAGGATCTTCACCTAGATCCTTTTAAATTAAAAATGAAGTTTTAAATCAATCTAAAGTATATATGAGTAAACTTGGTCTGACAGTTACCAATGCTTAATCAGTGAGGCACCTATCTCAGCGATCTGTCTATTTCGTTCATCCATAGTTGCCTGACTCCCCGTCGTGTAGATAACTACGATACGGGAGGGCTTACCATCTGGCCCCAGTGCTGCAATGATACCGCGAGACCCACGCTCACCGGCTCCAGATTTATCAGCAATAAACCAGCCAGCCGGAAGGGCCGAGCGCAGAAGTGGTCCTGCAACTTTATCCGCCTCCATCCAGTCTATTAATTGTTGCCGGGAAGCTAGAGTAAGTAGTTCGCCAGTTAATAGTTTGCGCAACGTTGTTGCCATTGCTACAGGCATCGTGGTGTCACGCTCGTCGTTTGGTATGGCTTCATTCAGCTCCGGTTCCCAACGATCAAGGCGAGTTACATGATCCCCCATGTTGTGCAAAAAAGCGGTTAGCTCCTTCGGTCCTCCGATCGTTGTCAGAAGTAAGTTGGCCGCAGTGTTATCACTCATGGTTATGGCAGCACTGCATAATTCTCTTACTGTCATGCCATCCGTAAGATGCTTTTCTGTGACTGGTGAGTACTCAACCAAGTCATTCTGAGAATAGTGTATGCGGCGACCGAGTTGCTCTTGCCCGGCGTCAATACGGGATAATACCGCGCCACATAGCAGAACTTTAAAAGTGCTCATCATTGGAAAACGTTCTTCGGGGCGAAAACTCTCAAGGATCTTACCGCTGTTGAGATCCAGTTCGATGTAACCCACTCGTGCACCCAACTGATCTTCAGCATCTTTTACTTTCACCAGCGTTTCTGGGTGAGCAAAAACAGGAAGGCAAAATGCCGCAAAAAAGGGAATAAGGGCGACACGGAAATGTTGAATACTCATACTCTTCCTTTTTCAATATTATTGAAGCATTTATCAGGGTTATTGTCTCATGAGCGGATACATATTTGAATGTATTTAGAAAAATAAACAAATAGGGGTTCCGCGCACATTTCCCCGAAAAGTGCCACCTGACGTC

1. **mApple-Control**

size:6225

cloning sites: 5’ – NotI & 3’ – XbaI

CMV promoter

KOZAK

FLAG-tag

mApple

bGH PA terminator

GACGGATCGGGAGATCTCCCGATCCCCTATGGTGCACTCTCAGTACAATCTGCTCTGATGCCGCATAGTTAAGCCAGTATCTGCTCCCTGCTTGTGTGTTGGAGGTCGCTGAGTAGTGCGCGAGCAAAATTTAAGCTACAACAAGGCAAGGCTTGACCGACAATTGCATGAAGAATCTGCTTAGGGTTAGGCGTTTTGCGCTGCTTCGCGATGTACGGGCCAGATATACGCGTTGACATTGATTATTGACTAGTTATTAATAGTAATCAATTACGGGGTCATTAGTTCATAGCCCATATATGGAGTTCCGCGTTACATAACTTACGGTAAATGGCCCGCCTGGCTGACCGCCCAACGACCCCCGCCCATTGACGTCAATAATGACGTATGTTCCCATAGTAACGCCAATAGGGACTTTCCATTGACGTCAATGGGTGGAGTATTTACGGTAAACTGCCCACTTGGCAGTACATCAAGTGTATCATATGCCAAGTACGCCCCCTATTGACGTCAATGACGGTAAATGGCCCGCCTGGCATTATGCCCAGTACATGACCTTATGGGACTTTCCTACTTGGCAGTACATCTACGTATTAGTCATCGCTATTACCATGGTGATGCGGTTTTGGCAGTACATCAATGGGCGTGGATAGCGGTTTGACTCACGGGGATTTCCAAGTCTCCACCCCATTGACGTCAATGGGAGTTTGTTTTGGCACCAAAATCAACGGGACTTTCCAAAATGTCGTAACAACTCCGCCCCATTGACGCAAATGGGCGGTAGGCGTGTACGGTGGGAGGTCTATATAAGCAGAGCTCTCTGGCTAACTAGAGAACCCACTGCTTACTGGCTTATCGAAATTAATACGACTCACTATAGGGAGACCCAAGCTGGCTAGTTAAGCTTGGTACCGAGCTCGGATCCACTAGTCCAGTGTGGTGGAATTCTGCAGATATCCAGCACAGTGGCGGCCgccaccATGgactacaaggacgatgacgacaagGGCGTGAGCAAGGGCGAGGAGAATAACATGGCCATCATCAAGGAGTTCATGCGCTTCAAGGTGCACATGGAGGGCTCCGTGAACGGCCACGAGTTCGAGATCGAGGGCGAGGGCGAGGGCCGCCCCTACGAGGCCTTTCAGACCGCTAAGCTGAAGGTGACCAAGGGTGGCCCCCTGCCCTTCGCCTGGGACATCCTGTCCCCTCAGTTCATGTACGGCTCCAAGGTCTACATTAAGCACCCAGCCGACATCCCCGACTACTTCAAGCTGTCCTTCCCCGAGGGCTTCAGGTGGGAGCGCGTGATGAACTTCGAGGACGGCGGCATTATTCACGTTAACCAGGACTCCTCCCTGCAGGACGGCGTGTTCATCTACAAGGTGAAGCTGCGCGGCACCAACTTCCCCTCCGACGGCCCCGTAATGCAGAAGAAGACCATGGGCTGGGAGGCCTCCGAGGAGCGGATGTACCCCGAGGACGGCGCCCTGAAGAGCGAGATCAAGAAGAGGCTGAAGCTGAAGGACGGCGGCCACTACGCCGCCGAGGTCAAGACCACCTACAAGGCCAAGAAGCCCGTGCAGCTGCCCGGCGCCTACATCGTCGACATCAAGTTGGACATCGTGTCCCACAACGAGGACTACACCATCGTGGAACAGTACGAACGCGCCGAGGGCCGCCACTCCACCGGCGGCATGGACGAGCTGTACAAGTAATCTAGAGGGCCCTTCGAACAAAAACTCATCTCAGAAGAGGATCTGAATATGCATACCGGTCATCATCACCATCACCATTGAGTTTAAACCCGCTGATCAGCCTCGACTGTGCCTTCTAGTTGCCAGCCATCTGTTGTTTGCCCCTCCCCCGTGCCTTCCTTGACCCTGGAAGGTGCCACTCCCACTGTCCTTTCCTAATAAAATGAGGAAATTGCATCGCATTGTCTGAGTAGGTGTCATTCTATTCTGGGGGGTGGGGTGGGGCAGGACAGCAAGGGGGAGGATTGGGAAGACAATAGCAGGCATGCTGGGGATGCGGTGGGCTCTATGGCTTCTGAGGCGGAAAGAACCAGCTGGGGCTCTAGGGGGTATCCCCACGCGCCCTGTAGCGGCGCATTAAGCGCGGCGGGTGTGGTGGTTACGCGCAGCGTGACCGCTACACTTGCCAGCGCCCTAGCGCCCGCTCCTTTCGCTTTCTTCCCTTCCTTTCTCGCCACGTTCGCCGGCTTTCCCCGTCAAGCTCTAAATCGGGGGCTCCCTTTAGGGTTCCGATTTAGTGCTTTACGGCACCTCGACCCCAAAAAACTTGATTAGGGTGATGGTTCACGTAGTGGGCCATCGCCCTGATAGACGGTTTTTCGCCCTTTGACGTTGGAGTCCACGTTCTTTAATAGTGGACTCTTGTTCCAAACTGGAACAACACTCAACCCTATCTCGGTCTATTCTTTTGATTTATAAGGGATTTTGCCGATTTCGGCCTATTGGTTAAAAAATGAGCTGATTTAACAAAAATTTAACGCGAATTAATTCTGTGGAATGTGTGTCAGTTAGGGTGTGGAAAGTCCCCAGGCTCCCCAGCAGGCAGAAGTATGCAAAGCATGCATCTCAATTAGTCAGCAACCAGGTGTGGAAAGTCCCCAGGCTCCCCAGCAGGCAGAAGTATGCAAAGCATGCATCTCAATTAGTCAGCAACCATAGTCCCGCCCCTAACTCCGCCCATCCCGCCCCTAACTCCGCCCAGTTCCGCCCATTCTCCGCCCCATGGCTGACTAATTTTTTTTATTTATGCAGAGGCCGAGGCCGCCTCTGCCTCTGAGCTATTCCAGAAGTAGTGAGGAGGCTTTTTTGGAGGCCTAGGCTTTTGCAAAAAGCTCCCGGGAGCTTGTATATCCATTTTCGGATCTGATCAAGAGACAGGATGAGGATCGTTTCGCATGATTGAACAAGATGGATTGCACGCAGGTTCTCCGGCCGCTTGGGTGGAGAGGCTATTCGGCTATGACTGGGCACAACAGACAATCGGCTGCTCTGATGCCGCCGTGTTCCGGCTGTCAGCGCAGGGGCGCCCGGTTCTTTTTGTCAAGACCGACCTGTCCGGTGCCCTGAATGAACTGCAGGACGAGGCAGCGCGGCTATCGTGGCTGGCCACGACGGGCGTTCCTTGCGCAGCTGTGCTCGACGTTGTCACTGAAGCGGGAAGGGACTGGCTGCTATTGGGCGAAGTGCCGGGGCAGGATCTCCTGTCATCTCACCTTGCTCCTGCCGAGAAAGTATCCATCATGGCTGATGCAATGCGGCGGCTGCATACGCTTGATCCGGCTACCTGCCCATTCGACCACCAAGCGAAACATCGCATCGAGCGAGCACGTACTCGGATGGAAGCCGGTCTTGTCGATCAGGATGATCTGGACGAAGAGCATCAGGGGCTCGCGCCAGCCGAACTGTTCGCCAGGCTCAAGGCGCGCATGCCCGACGGCGAGGATCTCGTCGTGACCCATGGCGATGCCTGCTTGCCGAATATCATGGTGGAAAATGGCCGCTTTTCTGGATTCATCGACTGTGGCCGGCTGGGTGTGGCGGACCGCTATCAGGACATAGCGTTGGCTACCCGTGATATTGCTGAAGAGCTTGGCGGCGAATGGGCTGACCGCTTCCTCGTGCTTTACGGTATCGCCGCTCCCGATTCGCAGCGCATCGCCTTCTATCGCCTTCTTGACGAGTTCTTCTGAGCGGGACTCTGGGGTTCGAAATGACCGACCAAGCGACGCCCAACCTGCCATCACGAGATTTCGATTCCACCGCCGCCTTCTATGAAAGGTTGGGCTTCGGAATCGTTTTCCGGGACGCCGGCTGGATGATCCTCCAGCGCGGGGATCTCATGCTGGAGTTCTTCGCCCACCCCAACTTGTTTATTGCAGCTTATAATGGTTACAAATAAAGCAATAGCATCACAAATTTCACAAATAAAGCATTTTTTTCACTGCATTCTAGTTGTGGTTTGTCCAAACTCATCAATGTATCTTATCATGTCTGTATACCGTCGACCTCTAGCTAGAGCTTGGCGTAATCATGGTCATAGCTGTTTCCTGTGTGAAATTGTTATCCGCTCACAATTCCACACAACATACGAGCCGGAAGCATAAAGTGTAAAGCCTGGGGTGCCTAATGAGTGAGCTAACTCACATTAATTGCGTTGCGCTCACTGCCCGCTTTCCAGTCGGGAAACCTGTCGTGCCAGCTGCATTAATGAATCGGCCAACGCGCGGGGAGAGGCGGTTTGCGTATTGGGCGCTCTTCCGCTTCCTCGCTCACTGACTCGCTGCGCTCGGTCGTTCGGCTGCGGCGAGCGGTATCAGCTCACTCAAAGGCGGTAATACGGTTATCCACAGAATCAGGGGATAACGCAGGAAAGAACATGTGAGCAAAAGGCCAGCAAAAGGCCAGGAACCGTAAAAAGGCCGCGTTGCTGGCGTTTTTCCATAGGCTCCGCCCCCCTGACGAGCATCACAAAAATCGACGCTCAAGTCAGAGGTGGCGAAACCCGACAGGACTATAAAGATACCAGGCGTTTCCCCCTGGAAGCTCCCTCGTGCGCTCTCCTGTTCCGACCCTGCCGCTTACCGGATACCTGTCCGCCTTTCTCCCTTCGGGAAGCGTGGCGCTTTCTCATAGCTCACGCTGTAGGTATCTCAGTTCGGTGTAGGTCGTTCGCTCCAAGCTGGGCTGTGTGCACGAACCCCCCGTTCAGCCCGACCGCTGCGCCTTATCCGGTAACTATCGTCTTGAGTCCAACCCGGTAAGACACGACTTATCGCCACTGGCAGCAGCCACTGGTAACAGGATTAGCAGAGCGAGGTATGTAGGCGGTGCTACAGAGTTCTTGAAGTGGTGGCCTAACTACGGCTACACTAGAAGAACAGTATTTGGTATCTGCGCTCTGCTGAAGCCAGTTACCTTCGGAAAAAGAGTTGGTAGCTCTTGATCCGGCAAACAAACCACCGCTGGTAGCGGTTTTTTTGTTTGCAAGCAGCAGATTACGCGCAGAAAAAAAGGATCTCAAGAAGATCCTTTGATCTTTTCTACGGGGTCTGACGCTCAGTGGAACGAAAACTCACGTTAAGGGATTTTGGTCATGAGATTATCAAAAAGGATCTTCACCTAGATCCTTTTAAATTAAAAATGAAGTTTTAAATCAATCTAAAGTATATATGAGTAAACTTGGTCTGACAGTTACCAATGCTTAATCAGTGAGGCACCTATCTCAGCGATCTGTCTATTTCGTTCATCCATAGTTGCCTGACTCCCCGTCGTGTAGATAACTACGATACGGGAGGGCTTACCATCTGGCCCCAGTGCTGCAATGATACCGCGAGACCCACGCTCACCGGCTCCAGATTTATCAGCAATAAACCAGCCAGCCGGAAGGGCCGAGCGCAGAAGTGGTCCTGCAACTTTATCCGCCTCCATCCAGTCTATTAATTGTTGCCGGGAAGCTAGAGTAAGTAGTTCGCCAGTTAATAGTTTGCGCAACGTTGTTGCCATTGCTACAGGCATCGTGGTGTCACGCTCGTCGTTTGGTATGGCTTCATTCAGCTCCGGTTCCCAACGATCAAGGCGAGTTACATGATCCCCCATGTTGTGCAAAAAAGCGGTTAGCTCCTTCGGTCCTCCGATCGTTGTCAGAAGTAAGTTGGCCGCAGTGTTATCACTCATGGTTATGGCAGCACTGCATAATTCTCTTACTGTCATGCCATCCGTAAGATGCTTTTCTGTGACTGGTGAGTACTCAACCAAGTCATTCTGAGAATAGTGTATGCGGCGACCGAGTTGCTCTTGCCCGGCGTCAATACGGGATAATACCGCGCCACATAGCAGAACTTTAAAAGTGCTCATCATTGGAAAACGTTCTTCGGGGCGAAAACTCTCAAGGATCTTACCGCTGTTGAGATCCAGTTCGATGTAACCCACTCGTGCACCCAACTGATCTTCAGCATCTTTTACTTTCACCAGCGTTTCTGGGTGAGCAAAAACAGGAAGGCAAAATGCCGCAAAAAAGGGAATAAGGGCGACACGGAAATGTTGAATACTCATACTCTTCCTTTTTCAATATTATTGAAGCATTTATCAGGGTTATTGTCTCATGAGCGGATACATATTTGAATGTATTTAGAAAAATAAACAAATAGGGGTTCCGCGCACATTTCCCCGAAAAGTGCCACCTGACGTC

1. **mApple-Myo10 CC mutant truncate**

Size: 9045 bp

cloning sites: 5’ – NotI & 3’ – XbaI

CMV promoter

KOZAK

FLAG-tag

mApple

linker

Hum MyoX (A938)+***I890A, L893Q, I897A, K904A point mutations***

bGH PA terminator

GACGGATCGGGAGATCTCCCGATCCCCTATGGTGCACTCTCAGTACAATCTGCTCTGATGCCGCATAGTTAAGCCAGTATCTGCTCCCTGCTTGTGTGTTGGAGGTCGCTGAGTAGTGCGCGAGCAAAATTTAAGCTACAACAAGGCAAGGCTTGACCGACAATTGCATGAAGAATCTGCTTAGGGTTAGGCGTTTTGCGCTGCTTCGCGATGTACGGGCCAGATATACGCGTTGACATTGATTATTGACTAGTTATTAATAGTAATCAATTACGGGGTCATTAGTTCATAGCCCATATATGGAGTTCCGCGTTACATAACTTACGGTAAATGGCCCGCCTGGCTGACCGCCCAACGACCCCCGCCCATTGACGTCAATAATGACGTATGTTCCCATAGTAACGCCAATAGGGACTTTCCATTGACGTCAATGGGTGGAGTATTTACGGTAAACTGCCCACTTGGCAGTACATCAAGTGTATCATATGCCAAGTACGCCCCCTATTGACGTCAATGACGGTAAATGGCCCGCCTGGCATTATGCCCAGTACATGACCTTATGGGACTTTCCTACTTGGCAGTACATCTACGTATTAGTCATCGCTATTACCATGGTGATGCGGTTTTGGCAGTACATCAATGGGCGTGGATAGCGGTTTGACTCACGGGGATTTCCAAGTCTCCACCCCATTGACGTCAATGGGAGTTTGTTTTGGCACCAAAATCAACGGGACTTTCCAAAATGTCGTAACAACTCCGCCCCATTGACGCAAATGGGCGGTAGGCGTGTACGGTGGGAGGTCTATATAAGCAGAGCTCTCTGGCTAACTAGAGAACCCACTGCTTACTGGCTTATCGAAATTAATACGACTCACTATAGGGAGACCCAAGCTGGCTAGTTAAGCTTGGTACCGAGCTCGGATCCACTAGTCCAGTGTGGTGGAATTCTGCAGATATCCAGCACAGTGGCGGCCgccaccATGgactacaaggacgatgacgacaagGGCGTGAGCAAGGGCGAGGAGAATAACATGGCCATCATCAAGGAGTTCATGCGCTTCAAGGTGCACATGGAGGGCTCCGTGAACGGCCACGAGTTCGAGATCGAGGGCGAGGGCGAGGGCCGCCCCTACGAGGCCTTTCAGACCGCTAAGCTGAAGGTGACCAAGGGTGGCCCCCTGCCCTTCGCCTGGGACATCCTGTCCCCTCAGTTCATGTACGGCTCCAAGGTCTACATTAAGCACCCAGCCGACATCCCCGACTACTTCAAGCTGTCCTTCCCCGAGGGCTTCAGGTGGGAGCGCGTGATGAACTTCGAGGACGGCGGCATTATTCACGTTAACCAGGACTCCTCCCTGCAGGACGGCGTGTTCATCTACAAGGTGAAGCTGCGCGGCACCAACTTCCCCTCCGACGGCCCCGTAATGCAGAAGAAGACCATGGGCTGGGAGGCCTCCGAGGAGCGGATGTACCCCGAGGACGGCGCCCTGAAGAGCGAGATCAAGAAGAGGCTGAAGCTGAAGGACGGCGGCCACTACGCCGCCGAGGTCAAGACCACCTACAAGGCCAAGAAGCCCGTGCAGCTGCCCGGCGCCTACATCGTCGACATCAAGTTGGACATCGTGTCCCACAACGAGGACTACACCATCGTGGAACAGTACGAACGCGCCGAGGGCCGCCACTCCACCGGCGGCATGGACGAGCTGTACAAGggcggccgaGATAACTTCTTCACCGAGGGAACACGGGTCTGGCTGAGAGAAAATGGCCAGCATTTTCCAAGTACTGTAAATTCCTGTGCAGAAGGCATCGTCGTCTTCCGGACAGACTATGGTCAGGTATTCACTTACAAGCAGAGCACAATTACCCACCAGAAGGTGACTGCTATGCACCCCACGAACGAGGAGGGCGTGGATGACATGGCGTCCTTGACAGAGCTCCATGGCGGCTCCATCATGTATAACTTATTCCAGCGGTATAAGAGAAATCAAATATATACCTACATCGGCTCCATCCTGGCCTCTGTGAACCCCTACCAGCCCATCGCCGGGCTGTACGAGCCTGCCACCATGGAGCAGTACAGCCGGCGCCACCTGGGCGAGCTGCCCCCGCACATCTTCGCCATCGCCAACGAGTGCTACCGCTGCCTGTGGAAGCGCCACGACAACCAGTGCATCCTCATCAGTGGTGAAAGTGGGGCAGGTAAAACCGAAAGCACTAAATTGATCCTCAAGTTTCTGTCAGTCATCAGTCAACAGTCTTTGGAATTGTCCTTAAAGGAGAAGACATCCTGTGTTGAACGAGCTATTCTTGAAAGCAGCCCCATCATGGAAGCTTTCGGCAATGCGAAGACCGTGTACAACAACAACTCTAGTCGCTTTGGGAAGTTTGTTCAGCTGAACATCTGTCAGAAAGGAAATATTCAGGGCGGGAGAATTGTAGATTATTTATTAGAAAAAAACCGAGTAGTAAGGCAAAATCCCGGGGAAAGGAATTATCACATATTTTATGCACTGCTGGCAGGGCTGGAACATGAAGAAAGAGAAGAATTTTATTTATCTACGCCAGAAAACTACCACTACTTGAATCAGTCTGGATGTGTAGAAGACAAGACAATCAGTGACCAGGAATCCTTTAGGGAAGTTATTACGGCAATGGACGTGATGCAGTTCAGCAAGGAGGAAGTTCGGGAAGTGTCGAGGCTGCTTGCTGGTATACTGCATCTTGGGAACATAGAATTTATCACTGCTGGTGGGGCACAGGTTTCCTTCAAAACAGCTTTGGGCAGATCTGCGGAGTTACTTGGGCTGGACCCAACACAGCTCACAGATGCTTTGACCCAGAGATCAATGTTCCTCAGGGGAGAAGAGATCCTCACGCCTCTCAATGTTCAACAGGCAGTAGACAGCAGGGACTCCCTGGCCATGGCTCTGTATGCGTGCTGCTTTGAGTGGGTAATCAAGAAGATCAACAGCAGGATCAAAGGCAATGAGGACTTCAAGTCTATTGGCATCCTCGACATCTTTGGATTTGAAAACTTTGAGGTTAATCACTTTGAACAGTTCAATATAAACTATGCAAACGAGAAACTTCAGGAGTACTTCAACAAGCATATTTTTTCTTTAGAACAACTAGAATATAGCCGGGAAGGATTAGTGTGGGAAGATATTGACTGGATAGACAATGGAGAATGCCTGGACTTGATTGAGAAGAAACTTGGCCTCCTAGCCCTTATCAATGAAGAAAGCCATTTTCCTCAAGCCACAGACAGCACCTTATTGGAGAAGCTACACAGTCAGCATGCGAATAACCACTTTTATGTGAAGCCCAGAGTTGCAGTTAACAATTTTGGAGTGAAGCACTATGCTGGAGAGGTGCAATATGATGTCCGAGGTATCTTGGAGAAGAACAGAGATACATTTCGAGATGACCTTCTCAATTTGCTAAGAGAAAGCCGATTTGACTTTATCTACGATCTTTTTGAACATGTTTCAAGCCGCAACAACCAGGATACCTTGAAATGTGGAAGCAAACATCGGCGGCCTACAGTCAGCTCACAGTTCAAGGACTCACTGCATTCCTTAATGGCAACGCTAAGCTCCTCTAATCCTTTCTTTGTTCGCTGTATCAAGCCAAACATGCAGAAGATGCCAGACCAGTTTGACCAGGCGGTTGTGCTGAACCAGCTGCGGTACTCAGGGATGCTGGAGACTGTGAGAATCCGCAAAGCTGGGTATGCGGTCCGAAGACCCTTTCAGGACTTTTACAAAAGGTATAAAGTGCTGATGAGGAATCTGGCTCTGCCTGAGGACGTCCGAGGGAAGTGCACGAGCCTGCTGCAGCTCTATGATGCCTCCAACAGCGAGTGGCAGCTGGGGAAGACCAAGGTCTTTCTTCGAGAATCCTTGGAACAGAAACTGGAGAAGCGGAGGGAAGAGGAAGTGAGCCACGCGGCCATGGTGATTCGGGCCCATGTCTTGGGCTTCTTAGCACGAAAACAATACAGAAAGGTCCTTTATTGTGTGGTGATAATACAGAAGAATTACAGAGCATTCCTTCTGAGGAGGAGATTTTTGCACCTGAAAAAGGCAGCCATAGTTTTCCAGAAGCAACTCAGAGGTCAGATTGCTCGGAGAGTTTACAGACAATTGCTGGCAGAGAAAAGGGAGCAAGAAGAAAAGAAGAAACAGGAAGAGGAAGAAAAGAAGAAACGGGAGGAAGAAGAAAGAGAAAGAGAGAGAGAGCGAAGAGAAGCCGAGCTCCGCGCCCAGCAGGAAGAAGAAACGAGGAAGCAGCAAGAACTCGAAGCCTTGCAGAAGAGCCAGAAGGAAGCTGAACTGACCCGTGAACTGGAGAAACAGAAGGAAAATAAGCAGGTGGAAGAG***gcC***CTCCGT***CaG***GAGAAAGAA***gcC***GAGGACCTGCAGCGCATG***gcc***GAGCAGCAGGAGCTGTCGCTGACCGAGGCTTCCCTGCAGAAGCTGCAGGAGCGGCGGGACCAGGAGCTCCGCAGGCTGGAGGAGGAAGCGTGCAGGGCGGCCTAATCTAGAGGGCCCTTCGAACAAAAACTCATCTCAGAAGAGGATCTGAATATGCATACCGGTCATCATCACCATCACCATTGAGTTTAAACCCGCTGATCAGCCTCGACTGTGCCTTCTAGTTGCCAGCCATCTGTTGTTTGCCCCTCCCCCGTGCCTTCCTTGACCCTGGAAGGTGCCACTCCCACTGTCCTTTCCTAATAAAATGAGGAAATTGCATCGCATTGTCTGAGTAGGTGTCATTCTATTCTGGGGGGTGGGGTGGGGCAGGACAGCAAGGGGGAGGATTGGGAAGACAATAGCAGGCATGCTGGGGATGCGGTGGGCTCTATGGCTTCTGAGGCGGAAAGAACCAGCTGGGGCTCTAGGGGGTATCCCCACGCGCCCTGTAGCGGCGCATTAAGCGCGGCGGGTGTGGTGGTTACGCGCAGCGTGACCGCTACACTTGCCAGCGCCCTAGCGCCCGCTCCTTTCGCTTTCTTCCCTTCCTTTCTCGCCACGTTCGCCGGCTTTCCCCGTCAAGCTCTAAATCGGGGGCTCCCTTTAGGGTTCCGATTTAGTGCTTTACGGCACCTCGACCCCAAAAAACTTGATTAGGGTGATGGTTCACGTAGTGGGCCATCGCCCTGATAGACGGTTTTTCGCCCTTTGACGTTGGAGTCCACGTTCTTTAATAGTGGACTCTTGTTCCAAACTGGAACAACACTCAACCCTATCTCGGTCTATTCTTTTGATTTATAAGGGATTTTGCCGATTTCGGCCTATTGGTTAAAAAATGAGCTGATTTAACAAAAATTTAACGCGAATTAATTCTGTGGAATGTGTGTCAGTTAGGGTGTGGAAAGTCCCCAGGCTCCCCAGCAGGCAGAAGTATGCAAAGCATGCATCTCAATTAGTCAGCAACCAGGTGTGGAAAGTCCCCAGGCTCCCCAGCAGGCAGAAGTATGCAAAGCATGCATCTCAATTAGTCAGCAACCATAGTCCCGCCCCTAACTCCGCCCATCCCGCCCCTAACTCCGCCCAGTTCCGCCCATTCTCCGCCCCATGGCTGACTAATTTTTTTTATTTATGCAGAGGCCGAGGCCGCCTCTGCCTCTGAGCTATTCCAGAAGTAGTGAGGAGGCTTTTTTGGAGGCCTAGGCTTTTGCAAAAAGCTCCCGGGAGCTTGTATATCCATTTTCGGATCTGATCAAGAGACAGGATGAGGATCGTTTCGCATGATTGAACAAGATGGATTGCACGCAGGTTCTCCGGCCGCTTGGGTGGAGAGGCTATTCGGCTATGACTGGGCACAACAGACAATCGGCTGCTCTGATGCCGCCGTGTTCCGGCTGTCAGCGCAGGGGCGCCCGGTTCTTTTTGTCAAGACCGACCTGTCCGGTGCCCTGAATGAACTGCAGGACGAGGCAGCGCGGCTATCGTGGCTGGCCACGACGGGCGTTCCTTGCGCAGCTGTGCTCGACGTTGTCACTGAAGCGGGAAGGGACTGGCTGCTATTGGGCGAAGTGCCGGGGCAGGATCTCCTGTCATCTCACCTTGCTCCTGCCGAGAAAGTATCCATCATGGCTGATGCAATGCGGCGGCTGCATACGCTTGATCCGGCTACCTGCCCATTCGACCACCAAGCGAAACATCGCATCGAGCGAGCACGTACTCGGATGGAAGCCGGTCTTGTCGATCAGGATGATCTGGACGAAGAGCATCAGGGGCTCGCGCCAGCCGAACTGTTCGCCAGGCTCAAGGCGCGCATGCCCGACGGCGAGGATCTCGTCGTGACCCATGGCGATGCCTGCTTGCCGAATATCATGGTGGAAAATGGCCGCTTTTCTGGATTCATCGACTGTGGCCGGCTGGGTGTGGCGGACCGCTATCAGGACATAGCGTTGGCTACCCGTGATATTGCTGAAGAGCTTGGCGGCGAATGGGCTGACCGCTTCCTCGTGCTTTACGGTATCGCCGCTCCCGATTCGCAGCGCATCGCCTTCTATCGCCTTCTTGACGAGTTCTTCTGAGCGGGACTCTGGGGTTCGAAATGACCGACCAAGCGACGCCCAACCTGCCATCACGAGATTTCGATTCCACCGCCGCCTTCTATGAAAGGTTGGGCTTCGGAATCGTTTTCCGGGACGCCGGCTGGATGATCCTCCAGCGCGGGGATCTCATGCTGGAGTTCTTCGCCCACCCCAACTTGTTTATTGCAGCTTATAATGGTTACAAATAAAGCAATAGCATCACAAATTTCACAAATAAAGCATTTTTTTCACTGCATTCTAGTTGTGGTTTGTCCAAACTCATCAATGTATCTTATCATGTCTGTATACCGTCGACCTCTAGCTAGAGCTTGGCGTAATCATGGTCATAGCTGTTTCCTGTGTGAAATTGTTATCCGCTCACAATTCCACACAACATACGAGCCGGAAGCATAAAGTGTAAAGCCTGGGGTGCCTAATGAGTGAGCTAACTCACATTAATTGCGTTGCGCTCACTGCCCGCTTTCCAGTCGGGAAACCTGTCGTGCCAGCTGCATTAATGAATCGGCCAACGCGCGGGGAGAGGCGGTTTGCGTATTGGGCGCTCTTCCGCTTCCTCGCTCACTGACTCGCTGCGCTCGGTCGTTCGGCTGCGGCGAGCGGTATCAGCTCACTCAAAGGCGGTAATACGGTTATCCACAGAATCAGGGGATAACGCAGGAAAGAACATGTGAGCAAAAGGCCAGCAAAAGGCCAGGAACCGTAAAAAGGCCGCGTTGCTGGCGTTTTTCCATAGGCTCCGCCCCCCTGACGAGCATCACAAAAATCGACGCTCAAGTCAGAGGTGGCGAAACCCGACAGGACTATAAAGATACCAGGCGTTTCCCCCTGGAAGCTCCCTCGTGCGCTCTCCTGTTCCGACCCTGCCGCTTACCGGATACCTGTCCGCCTTTCTCCCTTCGGGAAGCGTGGCGCTTTCTCATAGCTCACGCTGTAGGTATCTCAGTTCGGTGTAGGTCGTTCGCTCCAAGCTGGGCTGTGTGCACGAACCCCCCGTTCAGCCCGACCGCTGCGCCTTATCCGGTAACTATCGTCTTGAGTCCAACCCGGTAAGACACGACTTATCGCCACTGGCAGCAGCCACTGGTAACAGGATTAGCAGAGCGAGGTATGTAGGCGGTGCTACAGAGTTCTTGAAGTGGTGGCCTAACTACGGCTACACTAGAAGAACAGTATTTGGTATCTGCGCTCTGCTGAAGCCAGTTACCTTCGGAAAAAGAGTTGGTAGCTCTTGATCCGGCAAACAAACCACCGCTGGTAGCGGTTTTTTTGTTTGCAAGCAGCAGATTACGCGCAGAAAAAAAGGATCTCAAGAAGATCCTTTGATCTTTTCTACGGGGTCTGACGCTCAGTGGAACGAAAACTCACGTTAAGGGATTTTGGTCATGAGATTATCAAAAAGGATCTTCACCTAGATCCTTTTAAATTAAAAATGAAGTTTTAAATCAATCTAAAGTATATATGAGTAAACTTGGTCTGACAGTTACCAATGCTTAATCAGTGAGGCACCTATCTCAGCGATCTGTCTATTTCGTTCATCCATAGTTGCCTGACTCCCCGTCGTGTAGATAACTACGATACGGGAGGGCTTACCATCTGGCCCCAGTGCTGCAATGATACCGCGAGACCCACGCTCACCGGCTCCAGATTTATCAGCAATAAACCAGCCAGCCGGAAGGGCCGAGCGCAGAAGTGGTCCTGCAACTTTATCCGCCTCCATCCAGTCTATTAATTGTTGCCGGGAAGCTAGAGTAAGTAGTTCGCCAGTTAATAGTTTGCGCAACGTTGTTGCCATTGCTACAGGCATCGTGGTGTCACGCTCGTCGTTTGGTATGGCTTCATTCAGCTCCGGTTCCCAACGATCAAGGCGAGTTACATGATCCCCCATGTTGTGCAAAAAAGCGGTTAGCTCCTTCGGTCCTCCGATCGTTGTCAGAAGTAAGTTGGCCGCAGTGTTATCACTCATGGTTATGGCAGCACTGCATAATTCTCTTACTGTCATGCCATCCGTAAGATGCTTTTCTGTGACTGGTGAGTACTCAACCAAGTCATTCTGAGAATAGTGTATGCGGCGACCGAGTTGCTCTTGCCCGGCGTCAATACGGGATAATACCGCGCCACATAGCAGAACTTTAAAAGTGCTCATCATTGGAAAACGTTCTTCGGGGCGAAAACTCTCAAGGATCTTACCGCTGTTGAGATCCAGTTCGATGTAACCCACTCGTGCACCCAACTGATCTTCAGCATCTTTTACTTTCACCAGCGTTTCTGGGTGAGCAAAAACAGGAAGGCAAAATGCCGCAAAAAAGGGAATAAGGGCGACACGGAAATGTTGAATACTCATACTCTTCCTTTTTCAATATTATTGAAGCATTTATCAGGGTTATTGTCTCATGAGCGGATACATATTTGAATGTATTTAGAAAAATAAACAAATAGGGGTTCCGCGCACATTTCCCCGAAAAGTGCCACCTGACGTC

Movie S1. The Dynamic of filopodia induced by FL Myo10.

Movie S2. The Dynamic of filopodia induced by Myo10 HMM.

Movie S3. The Dynamic of filopodia induced by Myo10 CC mutant.
